# Review of the Common Deposition Methods of Thin-Film Pentacene, Its Derivatives, and Their Performance

**DOI:** 10.3390/polym14061112

**Published:** 2022-03-10

**Authors:** Yusniza Yunus, Nurul Adlin Mahadzir, Mohamed Nainar Mohamed Ansari, Tg Hasnan Tg Abd Aziz, Atiqah Mohd Afdzaluddin, Hafeez Anwar, Mingqing Wang, Ahmad Ghadafi Ismail

**Affiliations:** 1 Institute of Microengineering & Nanoelectronics, Universiti Kebangsaan Malaysia, Bangi 43600, Malaysia; P102853@siswa.ukm.edu.my (Y.Y.); P102852@siswa.ukm.edu.my (N.A.M.); hasnanaziz@ukm.edu.my (T.H.T.A.A.); a.atiqah@ukm.edu.my (A.M.A.); 2 Institute of Power Engineering, Universiti Tenaga Nasional, Bangi 43000, Malaysia; 3 Department of Physics, University of Agriculture, Faisalabad 38040, Pakistan; hafeez.anwar@gmail.com; 4 Institute for Materials Discovery, University College London, London WC1E 7JE, UK; mingqing.wang@ucl.ac.uk

**Keywords:** pentacene, thin-film deposition, solution-process, thermal vacuum evaporation, organic thin-film transistor, inkjet printing

## Abstract

Pentacene is a well-known conjugated organic molecule with high mobility and a sensitive photo response. It is widely used in electronic devices, such as in organic thin-film transistors (OTFTs), organic light-emitting diodes (OLEDs), photodetectors, and smart sensors. With the development of flexible and wearable electronics, the deposition of good-quality pentacene films in large-scale organic electronics at the industrial level has drawn more research attention. Several methods are used to deposit pentacene thin films. The thermal evaporation technique is the most frequently used method for depositing thin films, as it has low contamination rates and a well-controlled deposition rate. Solution-processable methods such as spin coating, dip coating, and inkjet printing have also been widely studied because they enable large-scale deposition and low-cost fabrication of devices. This review summarizes the deposition principles and control parameters of each deposition method for pentacene and its derivatives. Each method is discussed in terms of experimentation and theory. Based on film quality and device performance, the review also provides a comparison of each method to provide recommendations for specific device applications.

## 1. Introduction

Since the discovery of pentacene in 1912, many studies have been conducted over the decades to improve the conjugated organic molecule in terms of its solubility, stability, and sensitivity to oxygen and moisture [1,2]. Many pentacene precursors and substitutes have been made, and these pentacene derivatives had been synthesized and characterized for suitable usage in organic thin film transistors (OTFTs, this term is used interchangeably with organic field-effect transistor, OFET) [3,4], organic light emitting diodes (OLEDs) [5,6], and other organic electronic devices, such as thin-film sensors [7,8,9,10,11,12,13,14]. Pentacene is preferable due to its excellent semiconducting properties, being well understood and relatively cheap compared to the latest generation of organic semiconductors. With the newer improved pentacene derivatives, solution–process deposition has been made possible.

**Figure 1:**
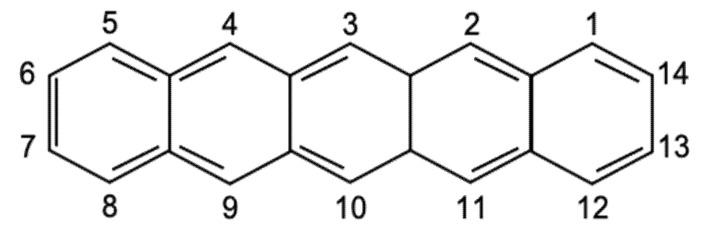
Carbon numbering of pentacene.

Pentacene itself contains five linearly fused aromatic rings and is also known as a polyaromatic hydrocarbon. Figure 1 shows an illustration of the flat molecules and carbon numbering of pentacene.

Unfortunately, pentacene is sensitive to ambient air (moisture and oxygen) and has low solubility, making it unstable when used with common fabrication techniques. Thus, pentacene should only be deposited using evaporation techniques. Initially, only ultra-high vacuum equipment was used in the deposition process for pentacene.

However, this technique is disadvantageous for industrial purposes as the process is expensive and does not cater for the fabrication of large-area electronics. High-temperature and high-vacuum conditions are needed in order to purify pentacene, which causes the volume to be degraded in the heat [2]. As pentacene is sensitive to oxygen, it is even more difficult to purify as contamination can occur quickly due to reaction with oxygen, meaning it must be carefully stored and treated in an inert atmosphere. It is also very damaging for pentacene to be exposed to water or UV light [2,15], as exposure has a huge influence on the mobility and the threshold voltage of organic transistors.

To overcome some of these issues, a treatment (out of the other various methods) involving self-assembled monolayers (SAMs) can be used to improve device performance. The use of SAMs and other treatments is widespread, although they are not extensively covered in this manuscript. In addition, an effective encapsulation process can also be applied to avoid exposure to oxygen and moisture. Boukhili et al. [15] provide further details on pentacene’s humidity effects. Pentacene and its derivatives are not new materials. Nevertheless, they are still widely used in research and prototyping, either alone or in conjunction (stacked or blended) with other compounds.

This manuscript is arranged into sections, first introducing pentacene and its derivatives followed by thin-film characterization. Next, the main sections cover the different deposition techniques. Within the main sections, there are a few treatments that are worth mentioning concurrently to explain the improvements that can be made in device performance. However, a focus is given to the rudimentary device fabrication techniques, without tapping into the more complex techniques, which may produce better-performing devices.

This manuscript provides background knowledge for readers who are unversed on the subject. It is aimed at young or more experienced researchers moving into a closely related or perhaps new field.

### 1.1. Pentacene Derivatives

The sensitivity of pentacene is due to its structure. This is because the diffusion of its five rings tempers the aromaticity and causes it to be more reactive [16]. This commonly occurs in the central ring, as the reaction of oxygen with the pentacene will produce an endo-peroxide on the central ring. Light and oxygen are the causes of this degradation and should be avoided during storage. These problems of solubility and stability were solved over the years through the development of two different methods [17].

**Figure 2:**
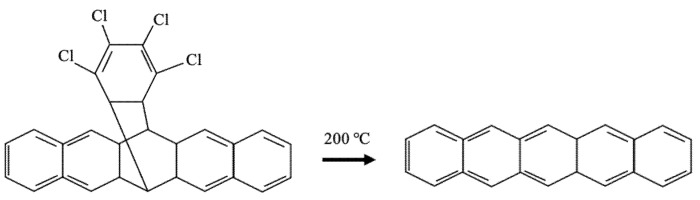
Example of a pentacene precursor structure.

The first method involves soluble pentacene precursors, as shown in Figure 2, which can be easily purified and deposited in solution form. This means that they are more soluble than the planar pentacene when deposited onto a substrate and can be converted into pentacene again using either heat or light. This process is called a retro-Diels–Alder reaction, which allows the precursors to be reconverted into semiconducting pentacene. This makes it competitive with other soluble semiconducting polymers, such as polypyrrole (PPy), polyaniline, and polydopamine (PDA).

**Figure 3:**
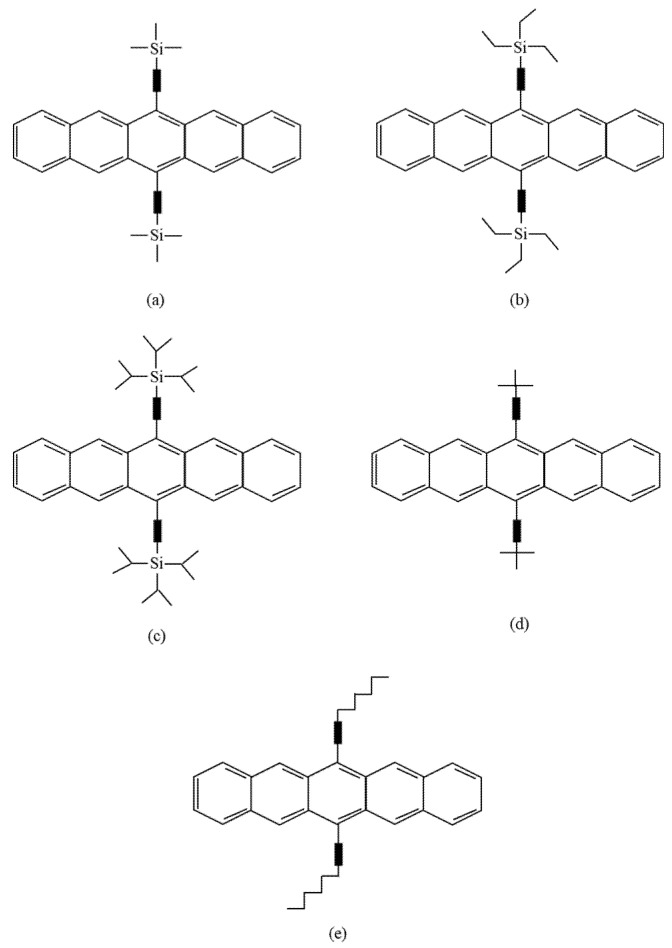
Structures of pentacene derivatives: (**a**) trimethylsilyl (TMS) pentacene; (**b**) triethylsilyl (TES) pentacene; (**c**) triisopropylsilyl (TIPS) pentacene; (**d**) t-butyl pentacene; (**e**) hexyl pentacene.

The second method involves introducing substituents onto the aromatic core (Figure 3), which increases the stability and solubility of the pentacene. It can also improve the electrical properties due to the self-assembly of pentacene moieties, which causes them to be packed closely together. This method has better purity control and allows solution processing. It also improves the stability of the original pentacene against oxygen [18,19]. However, these pentacene derivatives cannot be converted back into pentacene in the way pentacene precursors can.

The first pentacene precursor was proposed by Herwig et al. [20] in cooperation with Phillips in 1996 by adducting a dichloromethane solution into the pentacene. However, the heat required in this process is too high (~200 °C), making it incompatible with flexible substrates. The method was later improved by Weidkamp et al. [21] in 2002, whereby they prepared a precursor through the reaction of pentacene with a sulfinylamide in the presence of methyltrioxorhenium, which reduced the heating requirement to ~130 °C. They then produced a second precursor using a retro-Diels–Alder reaction via heating and UV lighting in the presence of a photoacid, whereby the non-converted precursor could be removed with methanol. Many precursors have been published since then to improve this method, as it helps to improve the solubility of the pentacene in the electronic fields.

The first pentacene derivative study was published in the year 2000, which focused on solving the solubility issues with polyacenes [22]. In 2001, Anthony et al. [23] studied the pentacene derivative of 6,13-bis(trimethylsilyethynyl)pentacene. Through this study, several well-known pentacene derivatives were discovered, such as TMS-pentacene, TES-pentacene, and TIPS-pentacene. This academic research approach was later adopted by Merck, as it meant pentacene derivatives could be purified via crystallization and column chromatography. It also significantly improved the stability issues against oxygen. However, this method has also shown degradation towards UV light, even without the presence of oxygen [24,25,26,27]. Additionally, the derivatives have an unfavorable influence on the electronic properties.

The importance of this discovery is to use soluble pentacene in organic solvents using solution-processable techniques such as spin coating and dip coating, as they are cheaper and easier to scale up. The high-solubility conjugated polymers are known to form homogenous films, which are important for obtaining large coverage areas and reproducible devices.

### 1.2. Common Thin-Film Pentacene Deposition Techniques

As the original pentacene is not solution-processable, the only available technique is the thermal vacuum evaporation technique, which is used to obtain continuous and reproducible films. This technique is expensive and difficult to scale up, since it requires high temperatures and very high vacuum pressures. However, it delivers high compound purity and good adhesion between the thin film compound and the substrate. It also provides easy control when depositing a very thin layer of material [2,15,28].

This solution-processable technique is widely studied and has been improved to produce common organic devices [29]. This technique reduces the fabrication costs and allows a broadened application of the devices on various substrates, such as flexible plastics and paper-based materials [30]. The spin coating and dip coating methods both have their advantages and disadvantages, depending on the solution and substrates being used [31].

### 1.3. Thin-Film Pentacene Characterization

There are three types of analysis methods that are used to observe the performances and outcomes of thin films—structural, optical, and electrical characterization methods.

Structural analysis approaches such as atomic force microscopy (AFM) are commonly used for topography imaging with ultra-small dimensions on any material surfaces, as well as for structural assessments of biological molecules, cellular components, cells, and tissues. This makes it suitable for viewing the surface morphology or topology of pentacene thin films [32,33]. This approach enables the identification of the physical characteristics of the materials, such as the roughness, as well as the electrical properties, such as the surface potential [34]. This allows the analysis of the deposited material’s molecular position and order, thereby helps to identify its effects on the device’s performance. Additionally, X-ray diffraction (XRD) is utilized to determine lattice mismatch between the thin film and substrate and to measure the stress and strain on the film caused by this lattice mismatch. It can also be used to determine the dislocation quality and density of the film by using rocking curve measurements and by measuring the lattice parameters in epitaxial multilayer structures. Additionally, XRD can also be used to determine the thickness, roughness, and film density. For thin-film characterization, it can help in identifying the crystallization that occurs within the thin film structure. As the main function of XRD is to identify phases presented in a crystalline material, it is commonly used to analyze pentacene deposition, as pentacene is known to have a crystalline structure [35,36].

Light absorption analysis approaches such as ultraviolet-visible spectroscopy (UV-Vis) are used to identify the material band gap based on the absorbance values obtained from the results. It can also be used to identify the compatibility of the pentacene thin-film material with the device’s structural layers in terms of flow through electrons and holes throughout the device structure [37,38].

As an example, the electrical characterization of thin-film pentacene transistors is usually performed to measure the field-effect mobilities, the on/off current ratio, the subthreshold slope, and the threshold voltage, depending on the kind of device being fabricated [39,40].

**Figure 4:**
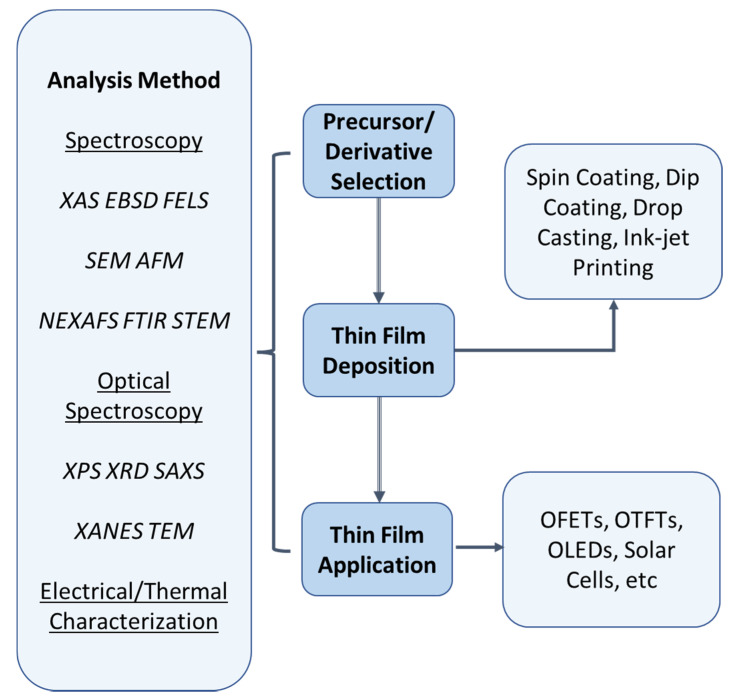
Soluble thin films—analysis, deposition, and applications.

Several other characterization approaches can be performed on deposited thin films based on the standard thin-film deposition process [41,42,43]. Figure 4 shows a summary of the soluble thin-film processes.

## 2. Thermal Vacuum Evaporation Methods

Thermal vacuum evaporation techniques are employed when the organic semiconductor can be sublimed, which is common for most small-molecule semiconductors [44,45]. Some examples of these techniques are organic molecular beam deposition (OMBD) [46] and organic vapor-phase deposition (OVPD) [31].

**Figure 5:**
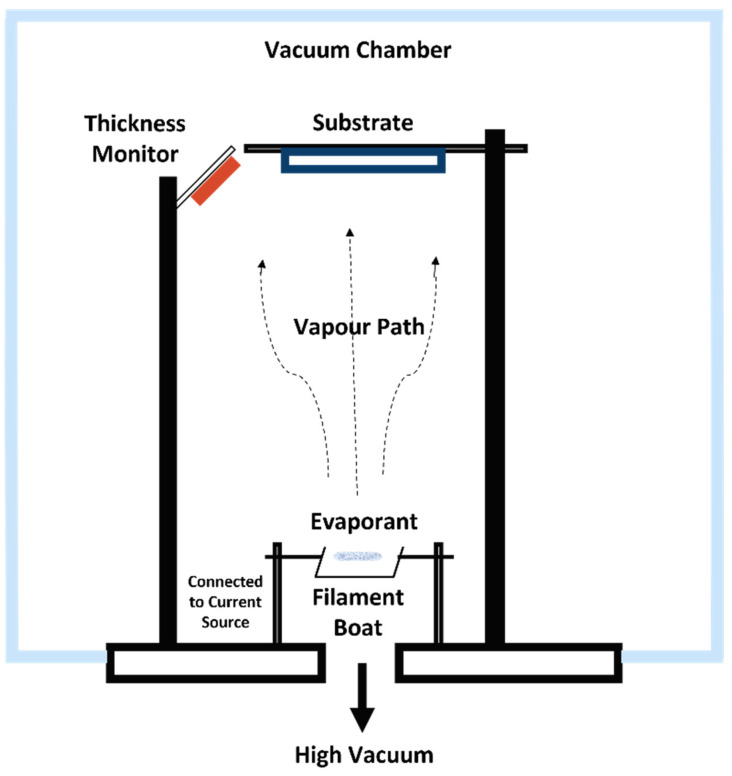
Thermal vacuum evaporation method.

For molecules such as pentacene, the OMBD technique is frequently used [47,48,49]. The technique uses a high to ultra-high vacuum (10^−6^–10^−12^ Torr) to evaporate the material (evaporant). The substrate is be placed parallel to the sublimed molecules inside a vacuum chamber and a semiconductor layer is formed on the substrate. The advantage of this technique is the efficient control of the growth conditions of the molecule film on the substrate. This means that the thin film produced on the substrate is in a well-ordered condition. The common thermal vacuum evaporation method is illustrated in Figure 5.

**Figure 6:**
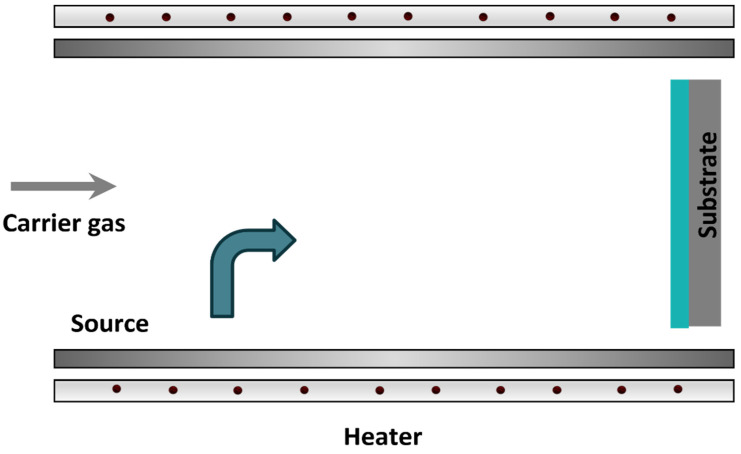
Organic vapor-phase deposition.

The OVPD technique, however, uses a relatively low vacuum pressure combined with a carrier gas [50,51]. In this technique, the material is evaporated and the carrier gas transports the molecules out through an outlet and towards the substrate that has been placed underneath. This technique is illustrated in Figure 6.

In 2003, Sheraw et al. [52] fabricated OTFTs with five different pentacene derivatives (TMS-pentacene, TES-pentacene, TIPS-pentacene, t-butyl-pentacene, and hexyl-pentacene) on a highly doped silicon substrate. The substrate temperature was varied from 30 to 60 °C and the nominal rate of the thermal evaporation was 0.1–0.5 Å s^−1^, while the system’s base pressure range was 2–5 × 10^−5^ Pa. The hexyl-pentacene results showed no field-effect-controlled conductivity. However, for TMS-pentacene fabricated at 30 °C, a field-effect mobility of 10^−5^ cm^2^ V^−1^ s^−1^ was obtained, similar to the result obtained with TES-pentacene fabricated at 60 °C. TIPS-pentacene fabricated at 90 °C and t-butyl-pentacene fabricated at 60 °C displayed the best operation results, with a field-effect mobility of 10^−4^ cm^2^ V^−1^ s^−1^.

**Figure 7:**
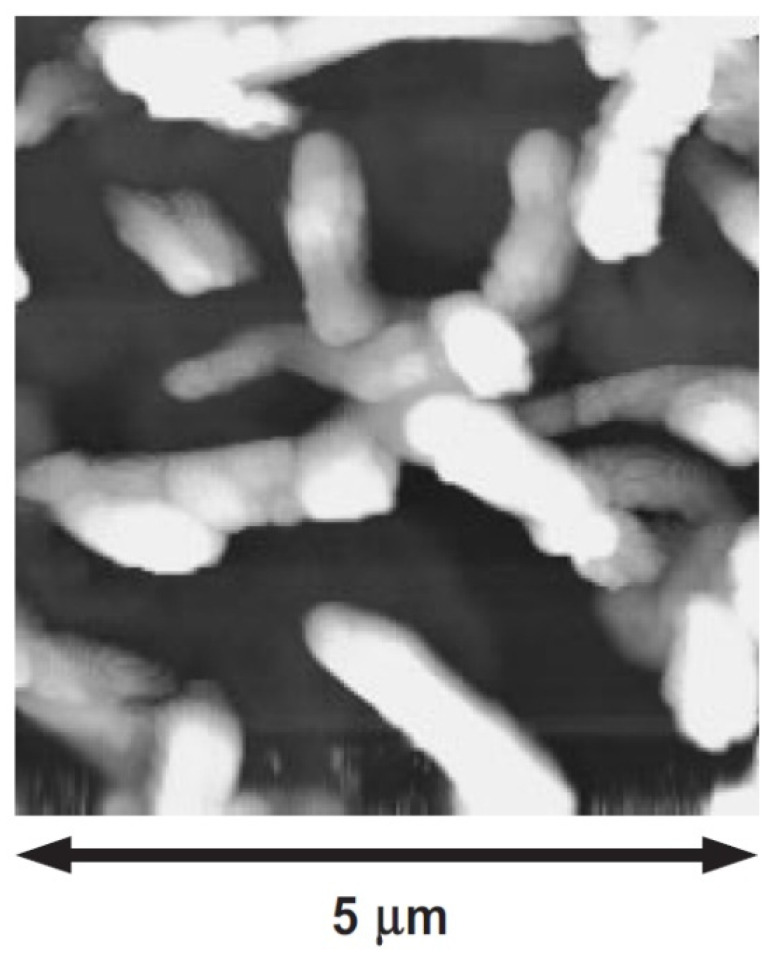
Grain formation of TIPS-pentacene thin film. Reprinted with permission from ref. [52]. 2013 John Wiley and Sons.

From these results, AFM was performed to observe the smooth morphology of the thin films of hexyl-pentacene, TMS-pentacene, and TES-pentacene, which led to low mobility. The reason for the low mobility was the amorphous formation of the films. The amorphous films cause the film to have a very smooth surface. Meanwhile, the surface morphology for TIPS-pentacene contained tall, long, thin grains and inadequate substrate coverage (Figure 7). The grain formation for TIPS and t-butyl thin films indicated the formation of polycrystalline grain structures.

Therefore, OTFTs based on TIPS-pentacene and t-butyl-pentacene on an octadecyl trichlorosilane (OTS)-treated substrate were fabricated. OTS is a silane coupling agent (SAM), meaning the treatment provides a low-surface energy substrate for organic layer growth and may reduce the tendency of the organic active layer and the silicon dioxide interface to hydrolyze [53,54]. The results from this treatment showed no improvement for t-butyl-pentacene. However, massive improvements were shown by TIPS-pentacene, with a field-effect mobility of 0.05 cm^2^ V^−1^ s^−1^.

The surface morphology of the TIPS-pentacene fabricated with the OTS-treated substrate was further improved by increasing the channel thin-film thickness from 50 nm to 75 nm, with a highly improved mobility of 0.4 cm^2^ V^−1^ s^−1^. This increased film thickness enlarged the grain size within the regions of continuous thin films between the grains.

This was the best field-effect mobility performance during that time. This gave us the knowledge that in order to obtain good performance, the surface morphology is important in defining the field-effect mobility. The substrate treatment also played an important role in this discovery. This shows that improvements can be made prior to the deposition of this material. Later, in 2007, Knipp et al. [28] reported a better-performing pentacene thin-film transistor deposited on a highly doped silicon wafer using the OMBD technique. Pure pentacene was used in this deposition process. Before the deposition process, the dielectric was treated with hexamethyldisilazane (HMDS) at a deposition rate of 0.5 Å s^−1^. The molecules were deposited at a base pressure of 5 × 10^−6^ Pa, with the substrate temperature kept constant at 70 °C.

A carrier mobility rate of 0.5 cm^2^ V^−1^ s^−1^ was observed, with a pentacene thickness of 10 nm. The subthreshold voltage was excellent at 0.2 V/decade. This is considered relatively low for an OTFT prepared on a 50 nm thermal oxide layer. This should have been two times higher than the obtained result, assuming that it was due solely to the gate capacitance. This section shows that over the years, new improvements have been made in the usage of pentacene.

Years later in 2012, Ochiai et al. [55] fabricated an OFET device using a polymer dielectric polycarbonate (PC). The pentacene was deposited in a vacuum with a pressure of 2 × 10^−4^ Pa and with the substrate temperature held at 50 °C. The deposition rate was set to 0.02 nm s^−1^, resulting in about 50 nm of pentacene thin film being formed.

The results showed a large mobility value of 0.62 cm^2^ V^−1^ s^−1^. This suggests the polycarbonate enhanced the crystallinity of the pentacene and increased the conductivity of the thin film. The high mobility was possibly due to the grain size and the microscopic molecular structures within the thin film, which significantly increased the charge transport properties. The efficient charge transport properties were highly influenced by the intermolecular interactions between the molecules, which were easily affected by the molecular packing and minimization of their reorganization energy.

In 2016, Saikia et al. [56] fabricated a bilayer anode of an OLED device consisting of fluorine-doped tin oxide (FTO) and pentacene. This was the first time pentacene was utilized as a buffer layer in OLED research.

**Figure 8:**
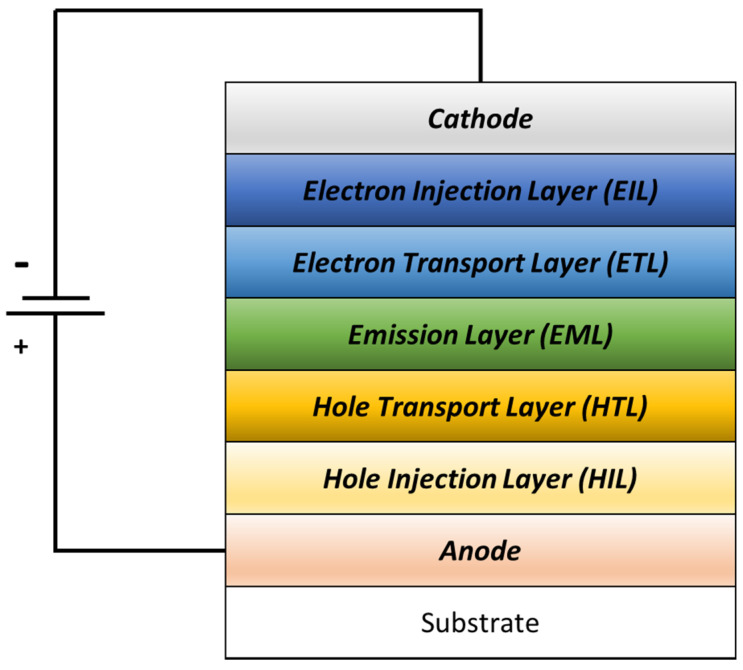
Schematic of the multilayers of an OLED structure.

The pentacene thicknesses were 1 nm, 4 nm, 6 nm, and 10 nm. The optimal thickness was found to be 6 nm, as this thickness increased the device’s current efficiency as well as the power efficiency compared to the bare FTO anode layer. A balanced contribution of the charge carrier’s thermionic emission was observed at this optimal thickness, which enhanced the current efficiency, as proven by the transmittance spectra and surface resistivity measurements. The obtained current efficiency was 6.6 Cd/A, which was higher than for the bare FTO at 3.7 Cd/A. In this case, pentacene was used to enhance the OLED performance. As pentacene is known for its high hole carrier mobility, it is commonly used as a hole injection layer. Figure 8 illustrates the OLED structure.

**Figure 9:**
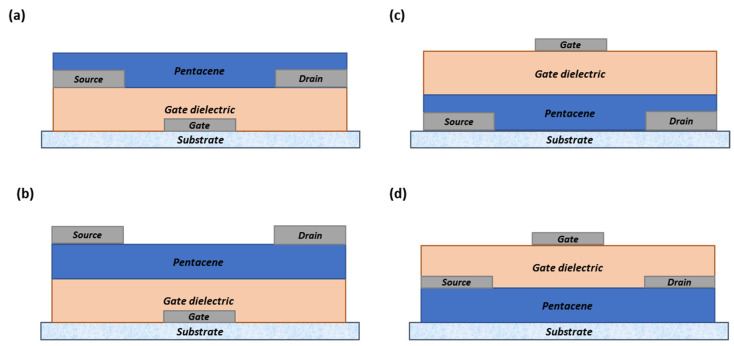
The common OTFT schematic structures: (**a**) bottom gate, bottom contact; (**b**) bottom gate, top contact; (**c**) top gate, bottom contact; (**d**) top gate, top contact.

In 2017, Lee et al. [24] studied the effects of the oxygen plasma flow rate generated during magnetron sputtering of ruthenium oxide (RuO_x_) on pentacene-based OTFTs. For this, different device structures were used, including bottom contact (BC) (Figure 9a,c) and top contact (TC) (Figure 9b,d) devices. TC devices are known to show better performance than BC devices due to the lower contact resistance between the source and drain electrodes and the reduced electron scattering at the semiconductor layer interface. The band alignment between the electrodes and the semiconductor layer is one of the keys to attaining good device performance. Note that pentacene has the highest occupied molecular orbital (HOMO) of 5.1 eV, while the work function of RuO_x_ is 4.92 eV.

The pentacene was deposited on a SAM-treated substrate measuring 50 nm in thickness using OMBD at 70 °C (substrate temperature) and 2 × 10^−6^ Torr with a deposition rate range of 0.2–0.3 Å s^−1^. The pentacene was patterned using a stencil mask. The oxidation of the deposited pentacene was measured using a UV tip cleaner with several exposure times of 30, 60, 120, and 180 min. When the magnetron sputtering deposited the RuOx electrodes, oxygen could have reacted with the pentacene thin films.

It was identified that there was no effect from the oxygen plasma on the BC devices; moreover, the device performance was found to be improve with the increase in oxygen gas flow rate. The mobilities were found to increase from 0.205 cm^2^ V^−1^ s^−1^ to 0.435 cm^2^ V^−1^ s^−1^. Similarly, with the TC device, as the oxygen flow rate increased, the field-effect mobilities increased from 0.308 cm^2^ V^−1^ s^−1^ to 0.499 cm^2^ V^−1^ s^−1^. However, in the TC device structure, the pentacene layer’s top surface was severely damaged and caused a leakage current. This also caused the leakage path to be comparable to the intrinsic channel and the devices eventually ceased operation overtime as the flow of the leakage current became more dominant when the flow of oxygen flow increased.

This is well-known phenomenon happens in polymers and small molecules when they interact with oxygen plasma. The acene rings in the molecular structure, which have a high carrier density, are easily destroyed by oxygen and ultraviolet (UV) light. They create new bonds, which act as carrier traps that degrade the device performance. The reaction with oxygen plasma in the TC device’s pentacene layer produced a derivative known as 6,13-pentacenequinone. The performance results for other thermally evaporated OTFTs are listed in Table 1 [55,57,58,59,60]. Note that the table lists only the average typical values relevant to this manuscript. The typical mobility rate for pristine pentacene OTFT is around 1 cm^2^ V^−1^ s^−1^; much higher mobility values have been reported in previous studies, such as from our previous work, which required more complex treatments with mobility rates of up to 4.7 cm^2^ V^−1^ s^−1^ [57].

**Figure 10:**
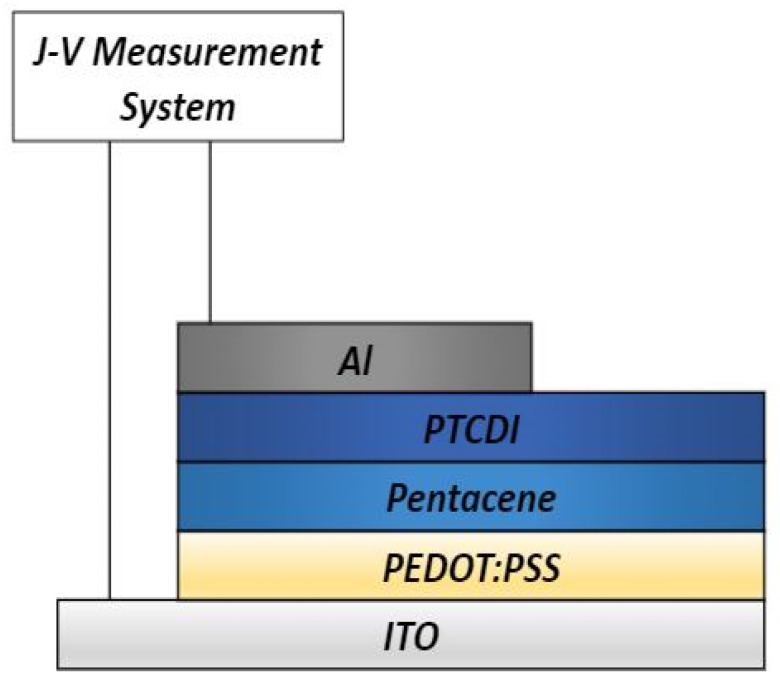
Illustration of the solar cell’s schematic structure.

Other than the common OFETs and OLEDs, pentacene was also fabricated onto solar cells. Biber et al. [82] fabricated a solar cell with the use of pentacene as part of their active layer. The pentacene was stacked with perylene tetracarboxylic di-imide (PTCDI) to form a donor–acceptor solar cell. This was performed by depositing the pentacene layer beforehand onto a PEDOT–PSS layer via thermal evaporation at a pressure of 3 × 10^−7^ Torr with a deposition rate of 1 Å s^−1^ to obtain 50 nm of pentacene. PTCDI measuring 50 nm in thickness was later deposited on top of the pentacene. Figure 10 shows an illustration of the solar cell structure.

The main objective was to determine the effects of the annealing time and temperature on the power conversion efficiency. Two groups of samples were fabricated. For the first group, the annealing temperatures were set to room temperature, 100 °C, and 150 °C for 5 min. For the second group, the annealing times were 5 and 10 min at a constant temperature of 100 °C.

The most important aspect in solar cells is the film morphology [82,83,84,85]. This can be observed through AFM images, as the morphology of the organic stacked layer is essential in analyzing a solar cell’s performance. In these images, the thin films that were annealed at high temperatures have a more homogeneous structure will show greater surface roughness compared to non-annealed or low temperatures. This is probably due to the increase in phase separation of the pentacene/PTCDI thin films, which allows an increase in the crystallization locations of the PTCDI.

The surface roughness is known to influence the charge carrier collection and the charge transport efficiency. Since the thin film’s roughness is decreased, this allows a larger contact area at the interface of the stacked materials, which enables it to boost the charge collection efficiency, giving a higher power conversion energy value. This is because high annealing temperatures activate the energy in the material’s atoms, leading to larger grain sizes.

However, this was only seen in the PTCDI layer, while the pentacene layer on the other hand showed decreased surface roughness with increases in both the annealing temperature and annealing time, which caused the efficiency of the solar cell to degrade. This was due to the reduction in charge collection, which increased the diffusion length of the excitons in the organic material and decreased the photocurrent. The combination of these layers did increase the light harvesting ability throughout the visible solar spectrum region. The decrease in the pentacene film’s surface roughness resulted in efficiency rates of 0.33%, 0.12%, and 0.06% for room temperature, annealing at 100 °C, and annealing at 150 °C, respectively [83].

Recently, pentacene layering for perovskite solar cells was investigated by Zhang et al. [86]. This study was performed to replace PEDOT–PSS as the hole transport layer in perovskite solar cells, as it is known to cause corrosion on the perovskite layer during the operation. Pentacene is seen as a good replacement due to its high hole mobility and the ease of its synthesis. Pentacene was deposited on an ITO substrate and the substrate temperature was varied between room temperature, 40 °C, 80 °C, and 120 °C.

**Figure 11:**
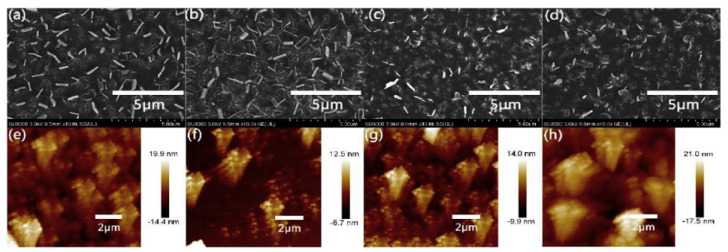
SEM images (**a**–**d**) and AFM images (**e**–**h**) of pentacene films prepared via thermoevaporation of pentacene on ITO substrates preheated at RT, 40 °C, 80 °C, and 120 °C, respectively. Reprinted with permission from ref. [86]. 2019 Elsevier.

Based on the XRD observation, the pentacene characteristic peaks under the preheated substrate at 40 °C were significantly narrower than the other conditions. The grain size of the pentacene crystallites was measured to be 70 nm, which was larger than the others. The pentacene thin film was further observed with SEM and AFM to study the surface morphology of the film (Figure 11). Under SEM observation, the pentacene morphology under the same preheated substrate conditions showed smaller holes on the surface and its crystallization increased vertically. This indicated that the crystallization is good for the perovskite–pentacene layer contact. The AFM observation of the same conditions showed that the pentacene thin film had high regularity and a lower surface roughness of 3.62 nm. The J-V measurement revealed that the solar cells exploiting the pentacene thin film produced a 5.7% power conversion efficiency (PCE) rate, which was much higher than the efficiency of the device based on bare ITO, with a 1.6% PCE rate. This shows that the pentacene thin film has a deep impact on the performance of perovskite solar cells.

Lubert-Perquel et al. [87] deposited pentacene with different deposition rates and discovered that the slower deposition rate was optimal for pentacene based on crystallization formation. However, pentacene forms better crystallites in mixed depositions. Thus, the study of pentacene in polymer blends is encouraging to improve the crystal growth of pentacene thin films [88,89].

In this study by Lubert-Perquel et al. [87], pentacene was co-evaporated with *p*-terphenyl, which was used to dilute pentacene in crystal and thin-film forms. A silicon wafer was used as the substrate. A 200 nm film was deposited onto the substrate at a base pressure of 3 × 10^−7^ mbar using OMBD. It was found that the ratio of the thin film to bulk phases was highly reliant to the orientation of the film. This study was aimed to provide a methodology for obtaining the desired orientation.

The addition of pentacene as the dopant in the *p*-terphenyl changed the thin film morphology and reduced the grain size by an order of magnitude. The higher the pentacene concentration, the smaller the grain size. The crystallites formed appeared to be more regular and the roughness was reduced from 93.5 nm to 16.5 nm. Since pentacene has polymorphism properties and grows in a mixed phase, its growth varies in temperature, thickness, and deposition rate. They achieved a pentacene thin film phase of d = 15.0 Å combined with a bulk phase of d = 14.1 Å. As the deposition rate increased, the thin film showed a lattice spacing with an isostructural increase of 0.1 Å. However, no phase separation was found when pentacene was mixed with *p*-terphenyl; it formed a homogenous blend instead, which enabled the gradual aggregation of the molecules to be investigated even further for use in organic devices. Other thermally evaporated optoelectronic device performance comparisons are listed in Table 2 [90,91].

It can be seen that the thermal vacuum evaporation method has been used in several studies for the deposition of pentacene. Other studies have also involved the implementation of this method with pentacene derivatives, such as, TMS-pentacene, TES-pentacene, TIPS-pentacene, t-butyl pentacene, and hexyl-pentacene, to observe their performances as active layers in OFET devices. Throughout these studies, it was clear that the film morphology is the main influence on the device performance. This led to the application of a surface treatment on the substrates prior to pentacene deposition. The commonly used surface treatments in these studies were OTS, HMDS, and PFDTES.

Beside the crystallinity, other factors requiring consideration are the temperature and surface conditions for pentacene deposition. Lassnig et al. [92] reported using three different surface conditions, namely sputtered, sputtered plus carbon, and unsputtered plus carbon, at sample temperatures of 200 K, 300 K, and 350 K during pentacene deposition to produce high-mobility pentacene transistors.

**Figure 12:**
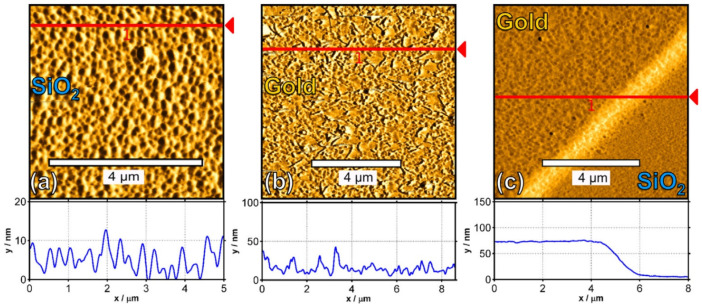
Pentacene deposition at 200 K: 1.5 monolayers on SiO_2_ (**a**) and gold (**b**), plus 7.5 monolayers on the gold contact–channel transition region (**c**). The corresponding cross-sections are indicated in the AFM scans by a line and shown underneath correspondingly. Reprinted with permission from ref. [92]. 2015 Elsevier.

**Figure 13:**
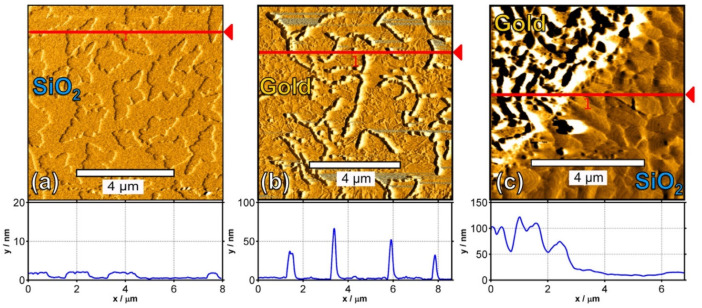
Pentacene deposition at 300 K: 1.5 monolayers on SiO_2_ (**a**) and gold (**b**), plus 7.5 monolayers on the gold contact–channel transition region (**c**). The corresponding cross-sections are indicated in the AFM scans by a line and shown underneath correspondingly. Reprinted with permission from ref. [92]. 2015 Elsevier.

**Figure 14:**
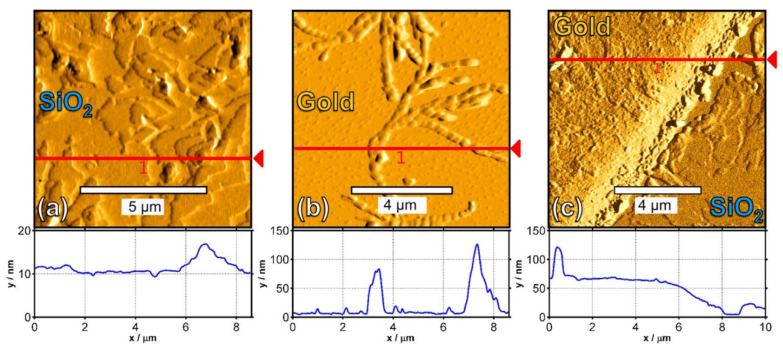
Pentacene deposition at 350 K: 7.5 monolayers on SiO_2_ (**a**) and gold (**b**), and on the gold contact–channel transition region (**c**). The corresponding cross-sections are indicated in the AFM scans by a line and shown underneath correspondingly. Reprinted with permission from ref. [92]. 2015 Elsevier.

AFM characterization was focused on the gold contact, the SiO_2_ along the channel region, and the critical gold/SiO_2_ transition region which revealed the underlying layer growth responsible for the electrical properties (Figure 12, Figure 13 and Figure 14).

Based on the AFM, Lassnig et al. [92] effectively combined the temperature and surface conditions for the pentacene deposition sequence using sputtering. The sequence consisted of four monolayers (ML) of pentacene deposited at 350 K as the layer within the channel with the most optimal charge transport features, followed by a four-ML covering layer deposited at 200 K, with optimal connection of the active layer to the gold electrodes. This method achieved a substantial carrier mobility increase compared to all other single-deposition temperature sputtering procedures.

It is worth mentioning that the effect of the polymer layer on the thin-film pentacene was also studied by depositing the polymer as a dielectric layer. It was found that the polymer caused the pentacene to form a good crystalline growth, which in turn improved the device carrier mobility [93,94]. Pentacene has also been assessed in other aspects by using this deposition method—pentacene was studied as an active layer, a buffer layer, and also as a dopant. This deposition method is not only applied in the fabrication of OFET devices but also in the fabrication of other devices, such as OLEDs and solar cells. For the thermal vacuum evaporation technique, devices are individually fabricated due to the limitations on space in the vacuum chamber. This deposition method is very costly, time-consuming, and not suitable for mass production. Hence, other deposition methods have been explored in order to solve these issues in the production process. The following alternative deposition methods, namely spin coating, dip coating, drop casting, and inkjet printing, are discussed in this review.

## 3. Spin Coating Method

**Figure 15:**
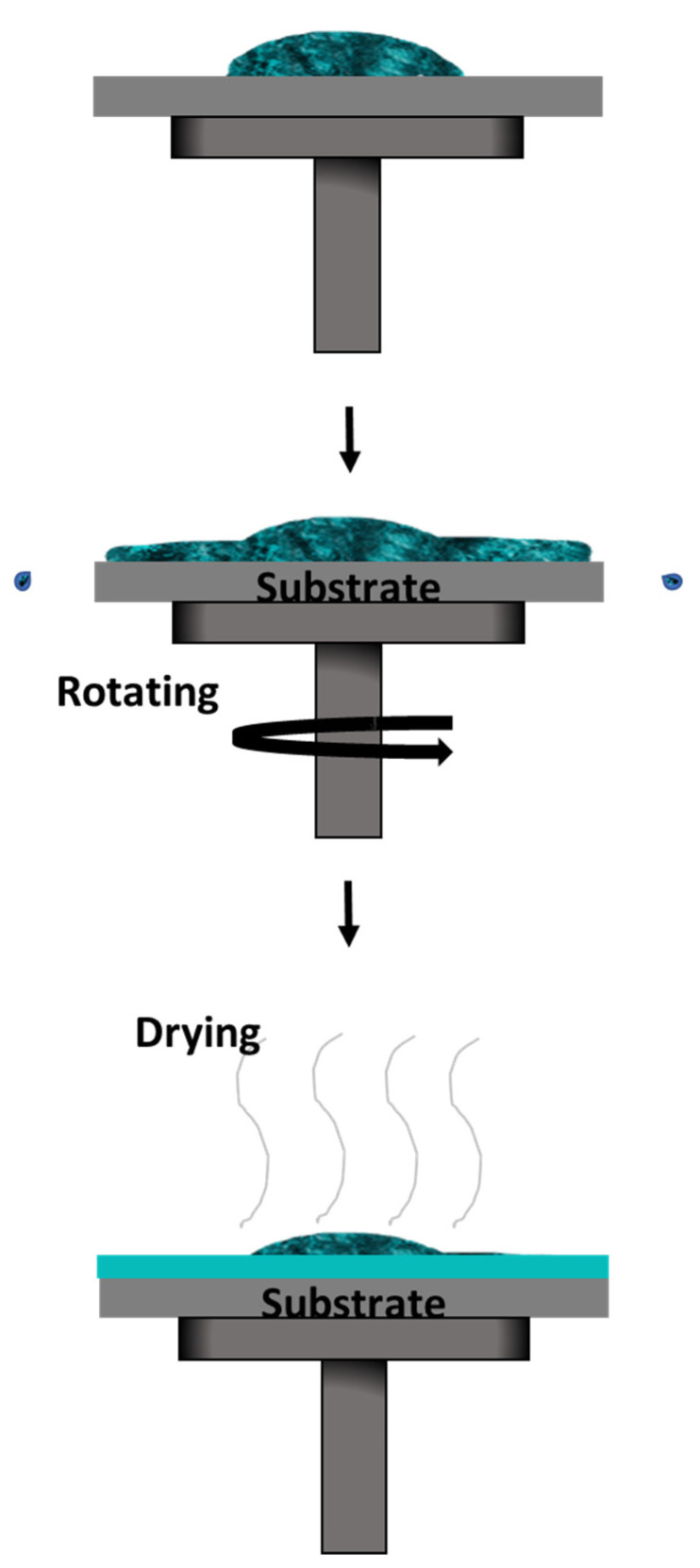
Spin coating method.

Spin coating is one of the most used methods in academic studies, as well as in industry, due to its capability for mass production at cheaper costs [95,96,97,98]. It is also easier to perform as it only requires a few drops of solution. The solution is dropped on top of a substrate that is held onto a chuck within the spin coater, as illustrated in Figure 15. The spinning process starts with a certain acceleration rate and remains at a constant speed for a period of time. Upon completion, the desired thin film will form via a combination of evaporation processes and liquid flow. An additional step such as heat or UV treatment may be required depending on the solution’s characteristics [31].

**Figure 16:**
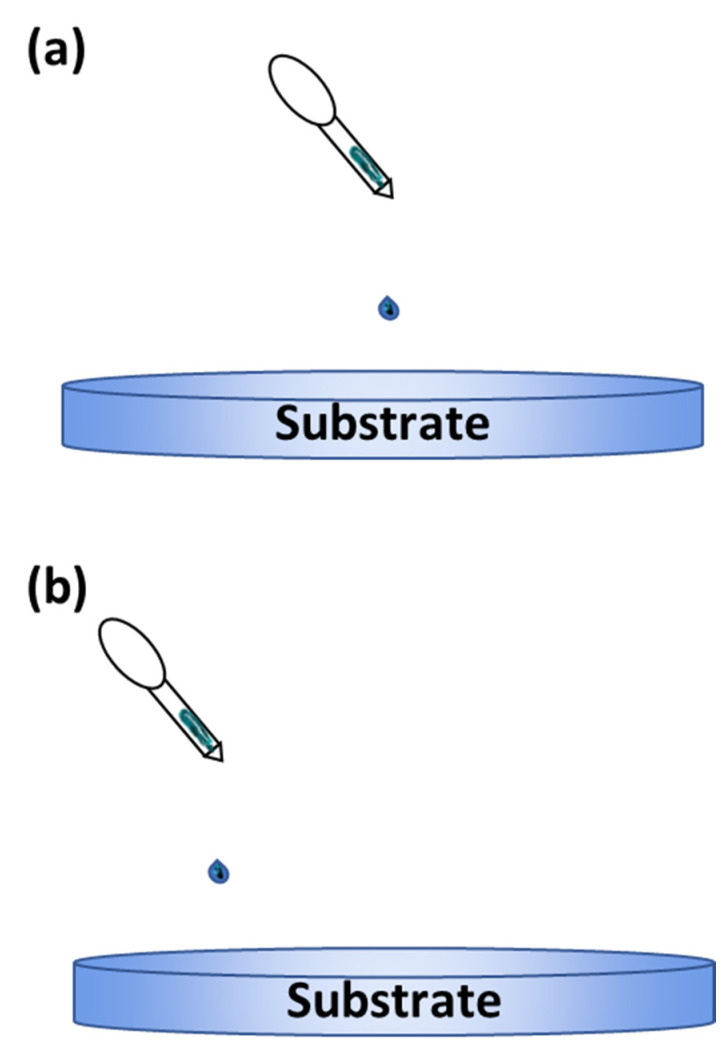
Illustration of the dropping positions: (**a**) central dropping; (**b**) off-centered dropping.

The surface coverage is important in this technique to ensure the uniformity of the thin film being deposited [99,100]. Bharti et al. [101] studied the deposition of TIPS-pentacene using this method with different dropping positions of the solution onto the substrate (Figure 16). They studied the effect it has on the alignment and crystallinity of the pentacene when it is deposited off-center to the substrate. Compared to the usual central dropping, the off-centered dropping approach was better for the crystallinity and pentacene alignment.

The thickness of the thin-film layer depends on the spin speed and duration of the spin. The solution’s properties, such as its density, viscosity, shear thinning, evaporation rates, and liquid flow, also effect the thickness. By altering the acceleration and spin speed, the liquid flow can be controlled. The evaporation rate on the other hand can majorly impact the morphology of the thin film. This method is suitably used to deposit pentacene precursors or pentacene derivatives, as they both are in solution form.

The solubility enables researchers to use pentacene with other soluble polymers in order to improve device performance. In 2016, Ozório et al. [61] produced a blend of P3HT–TIPS-pentacene as a thin film. The blended solution was deposited via spin coating onto two different Al_2_O_3_ insulators. One of the insulators was treated with HDMS while the other was not. A glass substrate was used to produce the OFET devices. They discovered that the crystalline growth in the thin film was better with the HDMS-treated Al_2_O_3_. This is highlighted because crystallinity it is important to determine the performance of the device.

For TIPS-pentacene, the carrier mobility depends greatly on the crystal orientation [102,103,104], crystalline thickness, crystalline domain size, and presence of cracks. Past research had showed a mobility rate of 1.8 cm^2^ V^−1^ s^−1^ for TIPS-pentacene transistors [105,106]. This result depends on the solution concentration, solvent selection, temperature of the thermal treatment, method of deposition, and substrate material. In general, it has been shown that solvents with a high boiling point deliver slow crystal growth. This enables the thin film to be formed with a large lamellar structure, possibly giving higher mobility.

Although heat treatment is needed for complete solvent evaporation, temperatures above 60 °C could damage the thin-film organic semiconductor. For this reason, it is not easy to obtain devices with uniform characteristics. Hence, polymer blends were developed to improve the morphology of the TIPS-pentacene. Doing so enabled control of the crystallinity of the film, grain size, and other parameters.

Ozório et al. [61] obtained a 1.2 × 10^−3^ cm^2^ V^−1^ s^−1^ mobility rate for the P3HT–TIPS-pentacene blend on an untreated oxide and 2.0 × 10^−3^ V^−1^ s^−1^ on a treated oxide. It was noted that the mobility could be higher with the presence of percolation of the crystalline domains as a continuous film along the surface. Moreover, the on/off current ratio was 10^2^ in both cases, with a 10^−9^ A rate for the off current. This value was relatively high due to the leakage in the oxide and not due to the carrier conduction in the semiconducting blend.

Subsequently, Ozório et al. [62] studied the solvent’s effects on the thin film morphology and the optical properties of the P3HT–TIPS-pentacene blend. Chloroform, toluene, and trichlorobenzene were used in their investigation by fabricating OFETs and using the P3HT–TIPS-pentacene blend as the active layer. An aluminum oxide substrate (Al_2_O_3_) was treated beforehand with HDMS. The semiconducting blend was mixed at a ratio of 1:1 (wt/wt) and dissolved separately in the three different solvents (chloroform, toluene, and trichlorobenzene). The boiling points of these solvents were 60 °C, 110 °C, and 200 °C, respectively. The prepared solutions were each filtered before being deposited by spin coating onto an Al_2_O_3_ layer at 1000 rpm for 60 s. The deposition was performed in a glovebox and the thickness produced was ~100 nm. The thin film was then annealed for 2 h in a vacuum oven at a temperature of 100 °C to remove the residual solvent.

**Figure 17:**
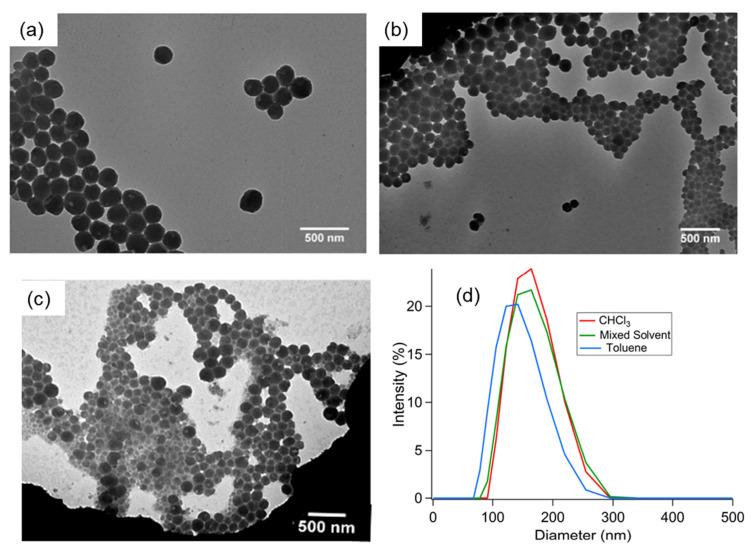
Representative TEM images of the P3HT nanoparticles synthesized from (**a**) chloroform, (**b**) toluene/chloroform (1:4 *v*/*v*), (**c**) toluene, and (**d**) intensity particle size distribution of P3HT nanoparticles obtained from DLS. Reprinted with permission from ref. [107]. 2012 American Chemical Society.

The TEM images for the thin film produced with the chloroform solvent showed that the TIPS-pentacene could be aggregated on the upper surface due to the phase difference (Figure 17). The high volatility of the solvent was found to be the cause of this. The high crystallization of the TIPS-pentacene was due to the solution’s saturation during the spin coating process as the solvent evaporated. This highly volatile solvent has a lower surface tension than the solute, which is also influenced by the spinner rotation, which means there is insufficient time for the film to organize properly, instead producing high surface roughness and structural defects [63,107,108].

The TEM images for films formed using toluene solvent show differences in the crystallites’ ordering compared to the film prepared using the chloroform solvent and the trichlorobenzene solvent. The film prepared with chloroform solvent shows “stripes” and “stretch marks” patterns of crystallites, while the film prepared with toluene solvent formed a smaller aggregated TIPS-pentacene with more “stretch marks” patterns of crystallites than the “stripes” pattern. However, the trichlorobenzene solvent’s thin film were seen to be more homogenous and less intense segregation. The crystalline aggregates were not observed in this thin film due to the low volatility of the solvent. This caused the solvent’s evaporation speed to be very low and delayed the solution’s saturation. Large aggregates of the TIPS-pentacene molecules were not formed on the surface when trichlorobenzene was used as the solvent. The molecule ordering in semiconducting polymetric materials has a considerable effect on the optoelectronic properties, such that the carrier mobility can be increased to a large degree by the molecule ordering. The UV-Vis spectra observations showed a narrower band gap for the thin film blends produced with toluene and trichlorobenzene. The device performances were observed based on the different solvents, namely chloroform, toluene, and trichlorobenzene, with mobility rates of 0.7 × 10^−3^ cm^2^ V^−1^ s^−1^, 1.0 × 10^−3^ cm^2^ V^−1^ s^−1^, and 5.0 × 10^−3^ cm^2^ V^−1^ s^−1^, respectively. The thin film blend produced with the trichlorobenzene solvent showed the best performance. This was due to the improved P3HT crystalline lamella ordering.

These results were observed in bottom gate, top contact device structures. It is believed that better performance could be obtained with top gate, top contact structures, as the current in the configuration was localized in an accumulated layer at the interface with the dielectric, while the molecules of the TIPS-pentacene were found to be concentrated on the upper surface. If the conduction was localized in the concentrated region of the TIPS-pentacene molecules, this could improve device performance.

The effect of crystallization on the TIPS-pentacene blended with an insulating polymer, polystyrene (PS), was studied by Madec et al. [105]. The solution blend was spin-coated onto a silicon substrate during the process of fabricating an OFET gas sensor. They observed the effects of the different spin coating duration on the crystallization of TIPS-pentacene, degree of phase separation, and field-effect characteristics of the thin film. The gas sensor characteristics were also affected by the spin coating duration.

This led to the discovery that vertical phase-separated structures were formed with TIPS-pentacene gathered on the top and PS gathered at the bottom, which caused a difference in the surface energy. Even though the vertical phase-separated structures were not influenced by the spin coating duration, the crystal growth of TIPS-pentacene molecules on the PS was still governed by it. A short spin coating time such as 3 s or 5 s would lead to 1D crystal growth, as it causes the excess residual solvent to induce convective flow in the drying droplet. Therefore, with a 50 s spin coating time, 2D crystal growth is achieved.

For comparison, the thin film with 1D crystal growth contained large-scale inter-crystal gaps, while the thin film with 2D crystal growth was found to be continuous with a high-density void. The field-effect mobility obtained from the 2D crystal growth thin film was ~0.6 cm^2^ V^−1^ s^−1^ and the on/off current ratio was 10^6^. It also exhibited a better sensing result compared to the 1D crystal growth, which had a field-effect mobility of ~0.3 cm^2^ V^−1^ s^−1^ and an on/off current ratio of 10^5^, which was due to the film thickness and the porous film structure. The 2D molecular crystals have a unique optoelectronic property that cannot be achieved in conventional bulk crystals. Thus, by utilizing their ultrathin structural features and superior interface qualities, one can enhance the performance of OFETs. Combining the intrinsic properties, such as the lightweight construction, material versality, and chemical and environment stability, would enable the 2D crystals to be used for advanced electronic technology applications [109].

It is known that the electrical performance of OTFTs is greatly dependent on the surface energy of the gate dielectric. Considering the active channel is located at the interface between the dielectric and semiconductor layers, a hydrophobic treatment of the gate dielectric surface can effectively increase the carrier mobility by several orders of magnitude. Therefore, it is crucial that the gate dielectric’s surface energy be lowered to improve the mobility. This would require a trade-off between the surface energy and surface wettability, making it challenging to produce a uniform thin film of an organic semiconductor on a hydrophobic dielectric (low surface energy) using a simple spin coating method.

Hence, two major approaches were taken. The first was to apply doping to change the surface tension of the organic semiconductor solution, thereby improving the surface wettability [61]. However, this doping had significant effects on the purity and the crystallinity of the semiconductor, directly impacting the mobility of the OTFT. The second was to dissolve the organic semiconductor in a solvent, which allowed the surface tension to be modified and enabled a continuous thin film to form on a hydrophobic dielectric. This approach was reported previously by Liu et al. [110], whereby OTFTs were fabricated by employing a low surface tension hexane as the solvent. A small-molecule thin film was deposited on the octadecyltrimethoxysilane (OTMS)-treated dielectric surface; however, the mobility was low at 0.02 cm^2^ V^−1^ s^−1^, albeit this showed an improvement in wettability. This report showed the importance of controlling the wettability of the organic semiconductor solution on hydrophobic surfaces to form uniform and continuous thin films.

In another related study, Wang et al. [64] investigated a low-viscosity organic semiconductor solution by spin coating a hydrophobic surface. Different solvents were tested to find the most suitable for producing a smooth, uniform, and continuous thin film. TIPS-pentacene was dissolved in hexane, toluene, chlorobenzene, 1,2-dichlorobenzene, and 1,2,3,4-tetrahydronaphthalene individually. Each solution was spin-coated onto an OTS/SiO_2_ substrate. They found that hexane produced the best results in terms of device performance. It improved the solution’s wettability by completely wetting the hydrophobic OTS/SiO_2_ surface. It also formed a continuous and uniform thin film. TIPS-pentacene samples prepared with hexane solution were further studied with different spin speeds and concentrations under ambient conditions, which were then annealed at 40 °C for 15 min. When the solution was dropped onto the substrate, a tri-phase contact line of the solution, substrate, and air was formed. In the beginning of the droplet evaporation, the evaporation-induced capillary flow forced the solute to transport to the tri-phase contact line region. The motion of the solute in the droplet was mainly influenced by the drag force and more solutes were transferred out to the tri-phase contact line, which then caused the solute–substrate Van der Waals and electrostatic interactions to increase. This resulted in contact line pinning and suggested that there was no surface tension. However, there was still a large effect of the surface tension when the tri-phase contact line was unpinned, as it extended the surface state between a solid and liquid. The surface tension of the hexane solution was 18.4 mJ m^−2^, which was approximate to the surface tension of the OTS/SiO_2_ substrate (18 mJ m^−2^). The continuous film was formed when the nucleation at the tri-phase contact line was produced due to the supersaturated solution.

There are three factors that affect the electrical properties: post-annealing, the spin coating speed, and the solution concentration. Post-annealing contributes to the enhancement of the field-effect carrier mobility. AFM images showed that the TIPS-pentacene morphology changed significantly after post-annealing at 40 °C for 15 min, while the RMS of the surface roughness was compared before and after the post-annealing, which resulted in a decrease from 18.7 nm to 2.37 nm. It was also noticed that the re-crystallized TIPS-pentacene became smooth and showed improved uniformity after post-annealing. The carrier mobility of the OTFT was shown to increase after the post-annealing from 0.12 to 1.66 cm^2^ V^−1^ s^−1^. This improvement was observed due to the better crystallinity of the thin film after the post-annealing process.

The second factor, the spin coating rotational speed, influences the carrier mobility. The solution was prepared using the hexane solvent with a 10 mg ml^−1^ TIPS-pentacene concentration. The morphologies and film thicknesses of the produced thin films were observed. When the spin speed was increased from 1000 rpm to 9000 rpm, the film thickness gradually decreased from 46 nm to 15 nm. Thinner films are formed at higher spin speeds due to the stronger centrifugal forces. All TIPS-pentacene films underwent the post-annealing process at 40 °C for 15 min. The mobility was seen to increasing as the speed increased from 1000 rpm to 7000 rpm, with 0.28 cm^2^ V^−1^ s^−1^ at 1000 rpm, 1.12 cm^2^ V^−1^ s^−1^ at 3000 rpm, 1.34 cm^2^ V^−1^ s^−1^ at 6000 rpm, and 1.66 cm^2^ V^−1^ s^−1^ at 7000 rpm. The thinner semiconducting layer reduced the parasitic resistance and improved the injection efficiency of the carriers [111,112]. However, when the spin speed reached 9000 rpm, the mobility reduced 0.85 cm^2^ V^−1^ s^−1^. Consequently, a film that is too thin can cause defects such as pin holes, grain boundaries, and trap states in the organic thin film.

The third factor, the solution concentration, is also known to influence the carrier mobility. The thin films’ morphologies and thicknesses produced with different solution concentrations of TIPS-pentacene at, 1, 4, and 10 mg ml^−1^ and prepared with hexane solvent were observed. The spin speed was set to 7000 rpm and all films were post-annealed at 40 °C for 15 min. It was observed that when the concentration of the solution increased, the film RMS surface roughness gradually decreased and the thickness of the thin film gradually increased. At 1 mg ml^−1^, the film RMS was 4.91 nm, with a thickness of 7 nm. The carrier mobility was merely 0.011 cm^2^ V^−1^ s^−1^. At 4 mg ml^−1^, the film RMS decreased to 3.26 nm with an increased thickness of 13 nm and a carrier mobility of 0.37 cm^2^ V^−1^ s^−1^. The concentration of 10 mg ml^−1^ gave the best results, with a film RMS of 2.37 nm, thickness of 22 nm, and carrier mobility of 1.66 cm^2^ V^−1^ s^−1^.

Wang et al. [64] obtained an important result for a solution-processed organic semiconductor thin film that was prepared in an ambient environment. The spin-coated organic solution’s low wettability on the hydrophobic OTS-treated SiO_2_ dielectric was effectively improved by using hexane as the solvent for the solution. The reduced surface tension contributed to the solution droplets’ increasing ability to adhere to the surface of the dielectric with low surface energy. The performance levels of other spin-coated OFETs are listed in Table 1, along with the ones discussed here.

The spin coating deposition method is one of the early solution-processable techniques. This allows materials such as pentacene to be deposited in solution form. The most commonly used soluble pentacene is TIPS-pentacene. It can be seen throughout this section that most research has been performed by spin coating TIPS-pentacene as the active layer. There are many important considerations when it comes to soluble processes—mainly the solution’s characteristics, such as its density, viscosity, evaporation rates, shear thinning, and liquid flow. This is where the choice of solvent and the solution concentration are important in determining these characteristics. Many have used solvents such as toluene, chloroform, chlorobenzene, and others in order to provide a uniform thin-film layer, as previously mentioned.

The morphology of the thin film is very important in producing good device performance. This includes the crystallinity growth of the soluble pentacene layer, leading to polymer blending studies. Polymer is known to improve the crystalline growth of pentacene. Thus, the solubility process allows them to be blended together as a single solution. Such blends have been observed and investigated based on the different ratios and concentrations between the TIPS-pentacene and the polymer. However, the common issues that can be seen throughout the blending are the non-uniformity of the deposited thin film and the influence of the thin film thickness on the carrier mobility. It is known that the thin film’s thickness depends on the spin speed and duration of the process. The spin coating deposition method is usually performed in a glove box in an inert environment, since it is vulnerable to contamination from humidity and oxygen, which can interact with the material when exposed to ambient air. When compared to thermal vacuum evaporation, the above are some of the disadvantages of the spin coating deposition process, although it is a cheaper alternative. To overcome these issues, the effects of the surface treatment have been studied when applied before the TIPS-pentacene deposition. Other alternative approaches to improve the uniformity of the film have also been studied, such as the implementation of a post-annealing process. This shows that the spin coating deposition method is not entirely suitable for all device fabrication scenarios due to the issues stated above. Further treatments need to be designed to obtain good device performances. Therefore, other solution-processable methods are also discussed in this review to compare and determine the suitability of these deposition methods with the desired device fabrication approaches.

## 4. Drop Casting Method

**Figure 18:**
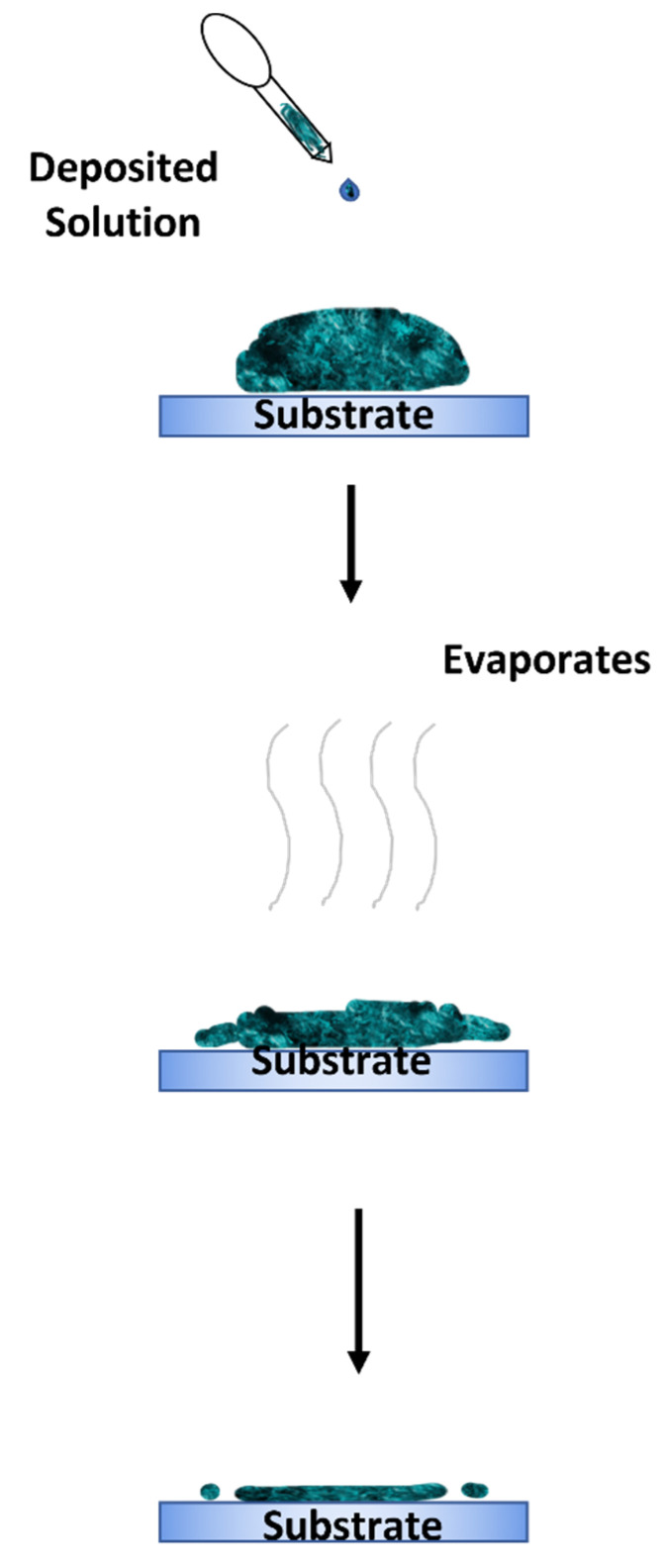
Drop casting method.

The drop casting method is an alternative deposition method that is more suitable for small-area deposition [113,114,115]. This method is performed by dropping a specified amount of solution on a static substrate and letting the solution evaporate for a desired amount of time. The substrate can also be baked to enhance the evaporation process. A solid thin film will then be formed on the substrate once the solution has dried up. The advantage of this method is its simple process. However, this method’s main disadvantages are the difficulty in obtaining a uniform and continuous coating on the deposited layer. The thickness of the deposited layer is also uncontrollable. Figure 18 shows an illustration of the drop casting method.

In 2016, Park et al. [116] used this deposition method to study terahertz modulation using a TIPS-pentacene thin film deposited on a patterned silicon substrate. The experiment was performed by drop casting 50 µL of TIPS-pentacene solution with a concentration of 2 mg/mL onto the substrate. It was then covered with a glass lid and heated on a 50 °C hotplate for 5 min. Despite the non-uniformity in the deposition process, the concentration of carriers injected into the TIPS-pentacene thin film rapidly became consistent over the whole area of the organic layer near the organic/inorganic interface. This was probably due to the fast in-plane diffusion of the carriers in the thin film itself.

The drop casting approach soon became known for producing semiconductors with a good crystallite structure, increasing device performance. Raghuwanshi et al. [67] investigated the crystal growth of TIPS-pentacene and its electrical stability on a flexible OFET upon bending. The tensile strain effect on the field-effect mobility was studied. TIPS-pentacene was mixed with toluene (1 wt.%) by stirring at 70 °C for 2.5 h. The solution was then deposited onto the substrate, which was tilted at an angle of ~5° and later covered with a glass Petri dish to maintain the substrate in a solvent-rich environment throughout the drying process. The substrate then underwent heat treatment at 80 °C to remove the residual solvent.

The solution was drop-casted on top of hafnium dioxide (HfO_2_) cross-linked with poly(4-vinylphenol) (PVP), as it offered a suitable surface for the TIPS-pentacene deposition, which resulted in a highly ordered arrangement for the crystal growth of the TIPS-pentacene. The device was operated at a low voltage of −15 V and showed excellent p-channel characteristics. It achieved a maximum carrier mobility of 0.12 cm^2^ V^−1^ s^−1^ and threshold voltages as low as −0.2 V. The device was conditioned to a tensile strain test with different bending radii for a period of 5 min, which resulted in a slight decrease in mobility and also an increase in the threshold voltage. The changes in the device performance were mainly caused by the changes in the dielectric morphology, as well as the surface roughness caused by the strain. Additionally, a disruption in uniformity at the semiconductor–dielectric interface also occurred, which was influenced by the magnitude of the strain rather than the duration. This indicated that the device had very good electrical stability under mechanical strain.

Since it is recognized that the crystallization of pentacene has a big influence on device performance, most investigations have been performed based on this assumption [117,118,119,120]. Blends of polymer solutions with pentacene solutions are considered to have the best potential to improve crystal growth. Asare-Yeboah et al. [121] studied the crystal growth of TIPS-pentacene with a poly(α-methyl styrene) (PαMS) blend to improve the performance of OTFTs. This investigation was performed with a temperature gradient technique to avoid the issues of the formation of random crystal orientations and poor areal coverage.

The TIPS-pentacene was mixed with PαMS in toluene, using 5 mg/mL of both. The blended solution was then drop-casted onto a substrate placed inside a Petri dish and sealed with parafilm. This was to allow the crystallization to occur in a solvent-rich environment. The crystal growth was facilitated by the slow solvent evaporation, which led to a large crystal size. By employing the temperature gradient technique, this not only improved the crystal alignment and enhanced the areal coverage but also eliminated the thermal cracks that were reported in the non-blended TIPS-pentacene thin film. This elimination enhanced the charge transport and increased the mobility to 10^−1^ cm^2^ V^−1^.

In 2017, Shih et al. [68] fabricated a solution-processable high-voltage organic thin-film transistor (HVOTFT). The use of high-voltage technologies at more than 100 V is not common in the organics field. This study was performed using TIPS-pentacene as the active layer, which was compared with a vacuum evaporation process for pure pentacene. A piranha-cleaned 100 mm diameter borosilicate glass plate was used as the substrate. In the solution process, TIPS-pentacene was dissolved in anisole at a concentration of 2 wt.%. It was then drop-casted in an ambient environment onto the substrate, which was angled at 4.5°. This process was performed over a 50 °C hotplate for 4–5 h. The substrate was covered while drying to provide a solvent-rich environment, as the solvent evaporates to form crystallization. It was then encapsulated with Perylene-C to ensure protection from moisture and chemical reactions.

The TIPS-pentacene-based HVOTFT showed varying results, which was probably caused by its semi-random crystal growth orientation. The breakdown voltage was 120 V and the carrier mobility was 0.005 cm^2^ V^−1^ s^−1^, which could be improved if larger crystal grains could be grown. When compared to the thermally deposited pentacene-based HVOTFT the results were better, with a breakdown voltage of 400 V and carrier mobility of up to 0.05 cm^2^ V^−1^ s^−1^. Hence, this study showed the possibility of flexible MEMS applications with the demonstrated solution-processed HVOTFT, with further improvements needed in terms of the crystal growth, crystal orientation, and grain size.

The following year, they improved the TIPS-pentacene-based HVOTFT by applying a SAM treatment in the fabrication process. The surface treatment was performed before the TIPS-pentacene was drop-casted, ss they had discovered in their previous study [69] that the leakage conduction paths in the bulk of the thin film were the cause of the poor I_ON_/I_OFF_ current ratio and low breakdown voltage. In order to improve this deficiency, the TIPS-pentacene layer thickness was lowered by coating the substrate with 1H,1H,2H,2H-perfluorodecyltriethoxysilane (PFDTES) SAM in a desiccator for 12 h. This process decreased the sample’s effective surface energy, which would help with the spreading of the TIPS-pentacene solution during the deposition process.

It was found that the surface treatment caused the solution to accumulate close to the lower part of the sample due to the low surface energy, which produced only a thin layer of TIPS-pentacene at approximately 100 nm on the majority of the surface. This was a huge difference compared to their previous study, whereby a ~1-µm-thick TIPS-pentacene layer was deposited. With this surface treatment they managed to obtain a high breakdown voltage exceeding 450 V. Additionally, an improved I_ON_/I_OFF_ current ratio and output characteristics were also achieved.

Concurrently, Raghuwanshi et al. [70] reported a blending system for an organic semiconductor and polymer for low-voltage flexible OFETs. TIPS-pentacene was used along with polystyrene as the polymer blend. Many things must be considered in this process, such as the deposition strategy, material properties of the polymer, the solvent used, and the mixing proportion, in order to obtain good electrical performance for the resulting OFET devices. Polystyrene is known as an insulating polymer that is able to form a phase-separated structure with small-molecule organic semiconductors. It does not disturb the TIPS-pentacene molecular bonds and can improve the uniformity of the thin layer morphology. It also provides good solubility in common organic solvents.

A flexible polyethylene terephthalate (PET) coated with ITO was used as the substrate. TIPS-pentacene (0.5 wt.%) and polystyrene were prepared separately in a toluene solvent. These solutions were stirred at 70 °C for 3 h and then a variety of blends were prepared. The TIPS-pentacene-to-polystyrene ratios were 3:1, 1:1, and 1:3 by volume, and the solutions were stirred for 30 min. The blended solutions were then drop-casted and the samples were covered with a Petri dish to provide a toluene-rich environment during evaporation. This whole process was performed in a dark room in ambient conditions.

Based on the results, the evaporation rate was slower with the higher polystyrene volume, which caused better crystallinity growth. The performance of the device increased with the increases in polystyrene in the blended ratio. The blended semiconductor ratios of 3:1, 1:1, and 1:3 showed maximum field-effect mobilities of 0.24, 0.25, and 0.57 cm^2^ V^−1^ s^−1^, respectively.

With increasing studies being performed on flexible devices, conformal OFETs have also entered into the research scope, with the potential to provide improvements to the current electronic devices, such as flexible displays, radio frequency identification tags, sensors, and logical circuits [122]. Zhou et al. [71] fabricated OFETs via drop casting a TIPS-pentacene solution onto a trichloro(phenyl)silane (PTS) dielectric, which was treated with an anti-solvent cross-linked poly(vinyl alcohol) (c-PVA). The structure was a bottom gate, top contact conformable OFET.

The fabrication started by dissolving TIPS-pentacene in chlorobenzene at a 0.1 wt.% concentration. This was then drop-casted onto the surface of the PTS-treated c-PVA layer with the substrate inclined. The substrate was baked at 50 °C under ambient condition to allow the crystallization process to occur.

The inclined drop casting method resulted in the growth of a vast, well-oriented single-crystal TIPS-pentacene microribbon array. This microribbon array had regular edges, a smooth surface, and high crystallinity. The array presented the best electrical results reported at that time, with a carrier mobility of 2.22 cm^2^ V^−1^ s^−1^. In addition, when the array was peeled off to become a conformed device, the carrier mobility was 0.87 cm^2^ V^−1^ s^−1^, which was excellent device performance for a conformable OFET.

Through the drop casting deposition method, the study of the application of pentacene solutions for device fabrication has broadened from conventional OFETs to electrolyte-gated OFETs (EGOFETs). Lago et al. [72] reported the use of TIPS-pentacene as a high-performance biocompatible electronic device that can operate in water. Organic semiconductors such as pentacene have attracted interest and have been implemented in new applications due to their fascinating properties, including their flexibility, transparency, and low-cost processability. This deposition method, which is more affordable and simple, makes device fabrication a lot easier and more cost-efficient. In their study, they aimed to develop a biocompatible sensor, focusing on the stability of the device operation when in prolonged contact with a strong saline solution.

TIPS-pentacene with a concentration of 0.5 wt.% was mixed with toluene. It was then drop-casted in ambient air at room temperature. The contact angle was approximately 100°, which showed the hydrophobicity of the deposited thin film. Even with such a simple deposition technique and the absence of a controlled environment during the deposition process, they obtained good performance from the fabricated device compared to the existing EGOFET devices that had been reported previously. The device operation could sustain a strong electrolyte at 37 °C and the growth of living cells was successfully accomplished directly on top of the device’s active layer. The device could also operate as a neural interface to recognize cells’ electrical activities.

Raghuwanshi et al. [73] expanded their studies on the blending of TIPS-pentacene and polystyrene in OFETs by again using a hybrid dielectric, although this time on a paper substrate. The emerging paper electronic field focuses on reusable and renewable devices. The use of paper has various advantages. It is known to be the most common resource used daily, with the benefits of being cheap, biodegradable, foldable, and with low roll-to-roll printing costs. However, its porous surface and high surface roughness restrict its usage in the fabrication of OFETs. A rough paper surface was planarized using a solution-processed polyvinyl alcohol (PVA) layer. The devices achieved a high carrier mobility rate of 0.78 cm^2^ V^−1^ s^−1^. The devices were observed to be highly stable during their operational period, with good performance compared to OFETs fabricated on glass and plastic. The performance levels of other drop-casted OFETs comparisons are listed in Table 1 [74,75].

Here, we review multiple flexible OTFTs and high-voltage OTFTs that were fabricated using the drop casting deposition method, along with discussing new and profound OFET structures. This deposition method is mostly performed to study the operational capability of such devices. The method is usually performed on a small substrate, avoiding the use of large amounts of materials, as the first step is usually just a draft of the device. Polymer blending has also been studied through this deposition method, which was found to produce high-quality crystalline growth. There is no other force that influences the deposited droplet; hence, the crystal growth orientation is only influenced by the substrate’s surface tension. Although it produces seemingly good crystal growth and is suitable for the fabrication of flexible and conformable devices, the thin film’s uniformity is not guaranteed. The non-uniformity of the thin film causes the carrier mobility of the device to be low and similar to that of spin-coated thin films. Furthermore, it is not suitable for mass production. Similar to spin coating, this technique is also very vulnerable to contamination from oxygen reactions with the material and humidity, which can damage the material unless the process is performed in an inert environment.

## 5. Dip Coating Method

**Figure 19:**
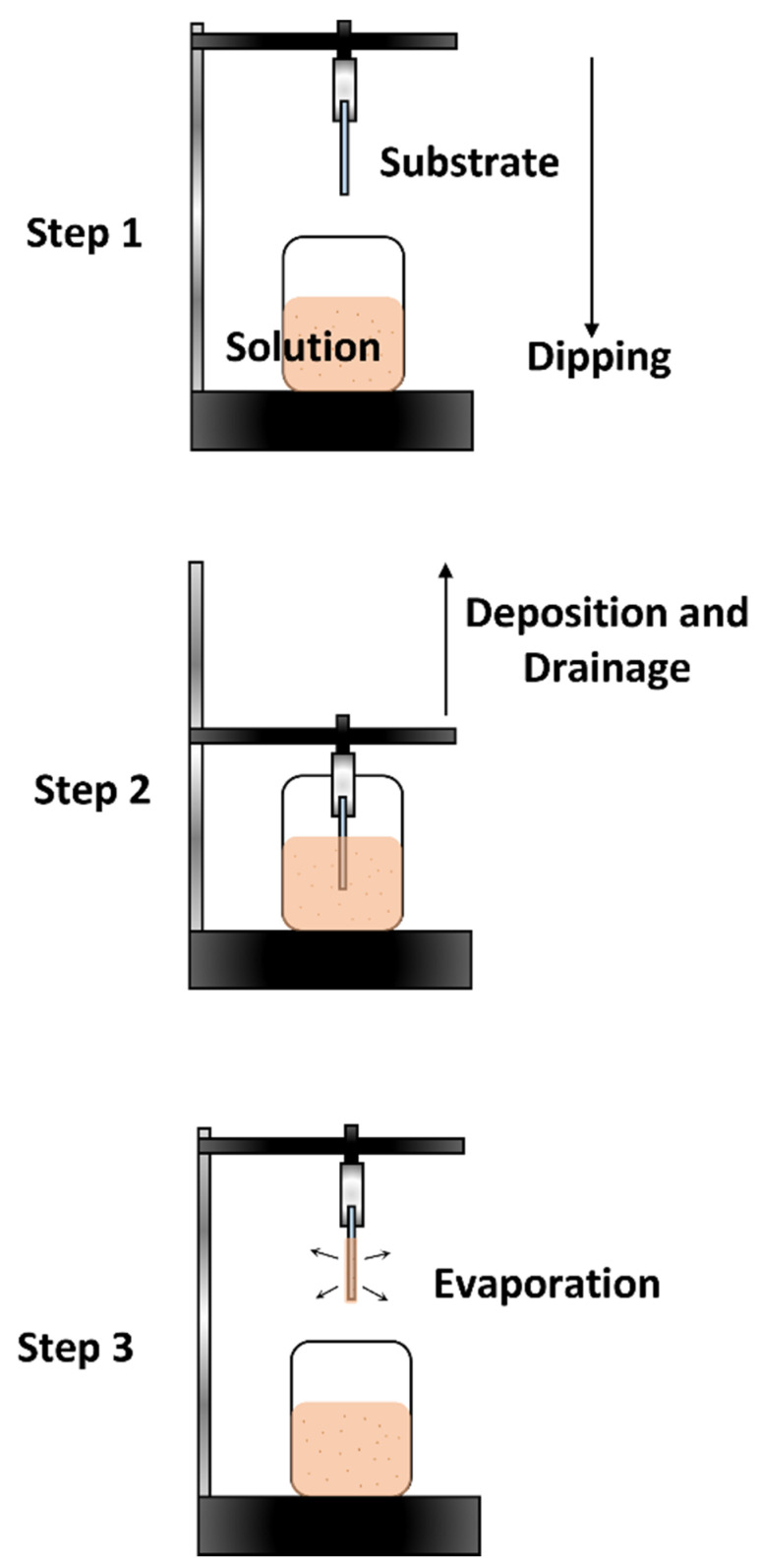
Dip coating method.

The dip coating method is also a commonly used technique, as it is fast and offers high uniformity when depositing thin-film layers [123,124,125]. The downside is that it requires a large amount of precursor solution in the reservoir. It is usually used to form thin films with complex and varying surface morphologies. It is conducted by suspending the substrate onto a clipper and then immersing it in the precursor solution below it. After a specified amount of time, the substrate will then be raised from the solution, enabling liquid film formation. It will then undergo an evaporation process that allows it to then form into a solid thin film. The thin film can then be processed further through thermal annealing or any other type of post-processing technique. Figure 19 shows an illustration of the dip coating method.

The increase or withdrawal speed of the substrate from the precursor solution determines the thickness of the thin-film layer. Other procedures can also be performed to alter the thickness of the thin film, such as varying the concentration of the precursor, the substrate temperature during the deposition, the acceleration rate, or even by performing the deposition at an angle [126,127,128,129]. However, it must be noted that several other forces influence the formation of the thin film, including the inertial forces, viscous drag, gravity, and the gradient of the surface tension [31].

The dip coating deposition allows researchers to study the possibility of depositing materials on rigid, flexible, and three-dimensional substrates, leading to a wide range of device development approaches for light-weight, flexible, and potentially low-cost devices. This has increased the interest in the deposition approach for large-area applications. In 2017, Wang et al. [76] fabricated a single-crystalline organic nanoribbon array on large-area OFETs. Usually, OFETs are made in smaller areas measuring ~10 cm^2^ in research labs. Instead, they fabricated OFETs in a large area measuring 50 cm^2^ by using the dip coating method. Bottom gate, top contact OFETs were constructed on silicon wafers with 300 nm of thermally grown SiO_2_ gate dielectric as the substrate. TIPS-pentacene was used as the active channel. A low boiling point solvent was preferred; thus, the solution was prepared using dichloromethane at a concentration of 4 mg mL^−1^. The withdrawal speeds in the dip coating process were 10, 30, 60, 80, and 120 μm s^−1^, in order to observe and obtain the optimum results for achieving high carrier mobility. This dip coating process was performed at room temperature.

The nanoribbons were observed to be deposited along the withdrawal direction as the dichloromethane solvent gradually evaporated. The nanoribbons formed in a continuous and well-aligned manner. The withdrawal speed at 80 μm s^−1^ was the most optimal for producing uniform and continuous crystal growth. The continuous crystal growth suggested the OFET device had high mobility. Nanoribbons measuring approximately 50 nm in thickness and 7–10 μm in width were obtained at this withdrawal speed, which had faceted edges with a ~1.2 nm smooth surfaces. Withdrawal speeds below 60 μm s^−1^ produced periodically aligned and short nanoribbons. At this rate, the TIPS-pentacene gradually accumulated at the contact line, which made the solution’s meniscus become too weighty, causing the depinning force to increase. This resulted in the formation of the short nanoribbon arrays. However, when the withdrawal speed was increased up to 120 μm s^−1^, non-continuous and defected nanoribbon arrays formed with lower crystallinity. It is known that the carrier mobility is highly affected by the crystallinity of the thin film. Other possible influences were further observed by varying the channel length from 10 to 200 μm. It was found that the carrier mobility gradually increased from 0.1 to 1.35 cm^2^ V^−1^ s^−1^ as the channel was lengthened [76]. The influence of the different metallic electrodes was also investigated by using electrodes such as gold (Au), silver (Ag), copper (Cu), and aluminum (Al). From this, researchers found that Cu exhibited better performance due to the valence band position of the HOMO at 5.37 eV [130,131,132]. Ag and Al were found to have a similarly low work function of ~4.2 eV, while the work function of Au was 5.1 eV. The working principle for Cu was more aligned with the working principle of TIPS-pentacene at 5.34 eV. This enabled them to combine the optimized configurations and achieve carrier mobilities as high as 3.2 cm^2^ V^−1^ s^−1^ [76].

The following year, Yang et al. [133] demonstrated a two-phase dip coating process in order to avoid the need for a large volume of solution simply by using a floating thin layer of solution on a reservoir of denser liquid, which did not form a homogenous mixture. This method requires strong wetting between the solvent and the substrate to promote the spreading of the solvent when it is withdrawn from the solution. This is also to ensure that the solution spreads continuously. TIPS-pentacene was used by mixing it with hexane at a concentration of 2 mg/mL. To start, a beaker was filled with 80% water and the substrate was partially submerged. As the substrate was withdrawn from the reservoir, the prepared solution was pipetted onto the water surface. The withdrawal speed was 1.2 cm h^−1^ and only 20 µL of solution was pipetted.

The results for this technique showed that the thin film yielded an oriented crystalline morphology with efficient charge transport along the long axis of the crystallites, with the hole mobility reaching 0.83 cm^2^ V^−1^ s^−1^. This technique was shown to be robust and required a minimal amount of material. It can also be completed in a short time with simplified instrumentation. Hence, it could be a preferrable method for low-cost applications.

It is clear that solution-processable pentacene can form crystalline and highly oriented polycrystalline films, which can influence the field-effect mobility [134]. However, in obtaining the field-effect mobility results, it must be noted that the bias dependency of the contact resistance of the source and drains electrodes on the channel can cause overestimation [135]. This is also due to the linearly aligned electrode arrangement, which produces a geometric mean of in-plane anisotropic mobilities in two-dimensional sheet conductors [136].

As mentioned above, it can be said that the dip coating deposition method does improve the continuity of the thin-film layer, which increases the uniformity of the thin film and results in less defects compared to the spin coating method. As discussed, this approach is preferable for various types of substrates that are flexible and irregular in shape, as this deposition method allows the solution to be spread over the substrate’s entire surface. The withdrawal speed is the main factor determining the thickness of the film. Other factors such as viscosity, liquid density, gravity, and surface tension can also influence the thickness. This is important, a as thin film’s thickness plays a role in enabling the carrier mobility flow. The withdrawal speed also influences the crystallinity growth of the thin film. Note that TIPS-pentacene was mainly used in the dip coating method. The crystalline growth of TIPS-pentacene thin films is important in producing a high carrier mobility rate. The optimum withdrawal speed and the type of solvent used when preparing the solution itself influence the formation of the thin film. This deposition method has shown much improvement as compared to the spin coating deposition method. However, this deposition method is not favorable due to the large volume of solutions used as the reservoir. This causes a lot of material waste, which conflicts with the aim of reducing the costs of the device fabrication process.

## 6. Inkjet Printing Method

The inkjet printing method is a widely used approach for digital printing, allowing high precision and control. During inkjet printing, droplets of ink with adjusted viscosity are propelled through a nozzle under piezoelectric or thermal force, which are deposited onto a substrate [137,138,139]. This technique can be applied with a wide range of materials. However, it also relies on the solution having sufficiently high surface tension while having sufficiently low viscosity to allow the solution to flow the through nozzle head only when required and to not leak uncontrollably. The quality of the solution is important in obtaining a uniform deposition.

**Figure 20:**
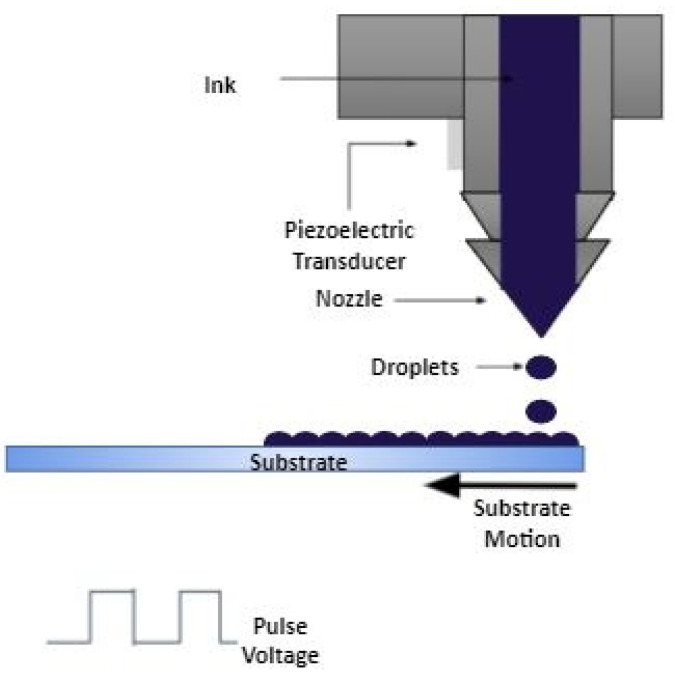
Inkjet printing method.

For piezoelectric inkjet printing, the solution is deposited according to the voltage pulse applied to the piezo transducer by distorting the nozzle and creating pressure to force out a controlled amount of solution. To stop the flow, the polarity of the voltage is reversed, expanding the nozzle. This inkjet printing deposition method is suitable for complex morphologies and arrays of materials [140,141,142,143,144,145]. Figure 20 shows an illustration of the inkjet printing method.

This method makes it possible for large-area deposition of various soluble materials. Since polymer blends have been known to overcome the difficulty of controlling crystal growth in soluble-processed TIPS-pentacene, as shown in previous studies [146], Cho et al. [78] investigated the behavior of TIPS-pentacene and amorphous polycarbonate (APC) in a polymer blend as the active layer using inkjet printing. Although the single-droplet inkjet printing of TIPS-pentacene with other polymer blends has been reported previously [147,148], the fabricated thin-film transistors showed issues in terms of film uniformity, especially when a geometrical parallel-type source drain was used, as the crystal orientation of the TIPS-pentacene in the single droplet was quite arbitrary.

Continuing from the study by Cho et al. [78], the active layer solution of TIPS-pentacene/APC was also compared with mixtures of TIPS-pentacene/PS and TIPS-pentacene/PαMS. The OTFT was fabricated on ITO substrates. The inkjet process was controlled with a head frequency of 200 Hz and 1000 DPI resolution. The print nozzle diameter was set to 30 μm. The inkjet printing was performed twice at 25 °C, then it was performed in succession with an interval of 3 s set by the printing system. The solvent was then air-dried and the deposited layers were annealed at 80 °C for 1 h on a hot plate in a nitrogen glove box.

The ratio of the blend was determined using the Flory–Huggins [149,150] and Cahn–Hilliard theories [151], whereby the Gibbs free energy when blending a polymer with TIPS-pentacene was evaluated. It is important to determine this ratio as it can affect the segregation strength between the TIPS-pentacene and the polymer phase. According to the theories, phase separation becomes easier with more positive Gibbs free energy values. In this study, the ratios were varied from 1:1 to 1:8 and were tested based on the OTFT performances. The highest mobility rate was obtained at 0.53 cm^2^ V^−1^ s^−1^ from the 1:4 ratio. This characteristic was observed to be closely related to the measurement of the phase separation strength between the TIPS-pentacene and the polymer phase. The crystal structures were then observed based on the various ratios. The crystals of inkjet-printed, non-blended TIPS-pentacene were highly anisotropic with various grain sizes, which led to a mobility rate of 0.22 cm^2^ V^−1^ s^−1^. The 1:1, 1:6, and 1:8 blended ratios produced plate-like wave crystalline structures with a random orientation, which resulted in carrier mobilities of 0.27 cm^2^ V^−1^ s^−1^, 0.35 cm^2^ V^−1^ s^−1^, and 0.17 cm^2^ V^−1^ s^−1^, respectively. Meanwhile, the 1:2 and 1:4 blended ratio produced stripe-shaped crystallite domains, resulting in carrier mobility rates of 0.46 cm^2^ V^−1^ s^−1^ and 0.53 cm^2^ V^−1^ s^−1^, respectively. This shows that the optimal TIPS-pentacene/APC mixing ratio induces strong phase separation and sequential crystal orientation, and it has a strong influence on the electrical properties of OTFTs.

The effect of the ink viscosity in wt.% on the device performance was examined [78]. The concentration was varied from 0.1 wt.% to 2.0 wt.% and it was found that the electrical performance of TIPS-pentacene/APC relied on the concentration as well as the viscosity of the inks. The average field-effect mobility seemed to increase from 0.04 cm^2^ V^−1^ s^−1^ to 0.53 cm^2^ V^−1^ s^−1^ when the concentrations were decreased from 2.0 wt.% to 1.0 wt.%. However, ink concentrations below 0.5 wt.% resulted in a lower mobility rate of 0.07 cm^2^ V^−1^ s^−1^ compared to the 1.0 wt.% and 1.5 wt.% concentrations at 0.53 cm^2^ V^−1^ s^−1^ (mentioned above) and 0.29 cm^2^ V^−1^ s^−1^, respectively. The inkjet-printed film with higher viscosity of 2.0 wt.% exhibited a broad and even distribution of TIPS-pentacene at all depth positions of the layer, with a slight occurrence of TIPS-pentacene at the top of the surface of the thin layer. The thin film with a 1.5 wt.% concentration showed a disconnected TIPS-pentacene layer in the top area, while at 1.0 wt.% the TIPS-pentacene was found to be discrete and showed a sharp phase separation in the upper area of the film. The OTFTs with the low-viscosity inks at 0.1 wt.% and 0.5 wt.% concentrations showed barely any distinguishable TIPS-pentacene layer at any position of the film, which meant that there were very small numbers of TIPS-pentacene crystals. Thus, ink viscosities lower than 0.5 wt.% does not produce a good OTFT performance.

Using the optimal ratio of 1:4, various solvents were tested for the TIPS-pentacene/APC solution, including toluene, toluene/chloroform, toluene/*p*-xylene, and toluene/tetralin. This was to investigate the effects of solvents on the field-effect mobility. The boiling point was the focus, as only 5 wt.% of the minor solvent was mixed with 95 wt.% of the major solvent (toluene), which was fixed. Toluene/*p*-xylene was shown to produce the highest mobility of 0.53 cm^2^ V^−1^ s^−1^ compared to toluene/chloroform, toluene, and toluene/tetralin, which exhibited carrier mobility rates up to 0.30, 0.31, and 0.40 cm^2^ V^−1^ s^−1^, respectively. This led to the observation of the thin films’ morphologies. It was shown that the Marangoni and convective flows of the drying ink are crucial for controlling the morphology of the inkjet droplets [152]. The high boiling point solvent, which was used as the major solvent, was mixed with a lower boiling point solvent, which was used as the minor solvent to control the evaporation rate of the droplets. As the evaporation rate increased at the edges of the droplets, this caused a buildup of convective flow from the center toward the drying edge of the droplets. This drove the surface tension gradient between the center and edge of the droplets. However, the opposing Marangoni flow arose in order to recirculate the solvents in the droplet. When the lower minor solvent boiling point was used, it produced a greater convective flow toward the drying edge, resulting in a higher edge wall of the droplet, which was known as the coffee ring effect. Thus, in this case, where a higher minor solvent boiling point was used, a weaker convective flow was created, with a smaller coffee ring effect. This behavior of the inkjet droplets influenced the morphology of the final printed thin film. Better-oriented TIPS-pentacene crystals were produced from the toluene/*p*-xylene solvents compared to the others. This also resulted in non-oriented large grain boundaries.

Finally, a comparison was made using different polymers in the blending system, namely PS and PαMS. These TIPS-pentacene polymer blends were inkjet-printed and resulted in carrier mobility rates of 0.2 and 0.34 cm^2^ V^−1^ s^−1^ for TIPS-pentacene/PS and TIPS-pentacene/PαMS, respectively, which were lower than for TIPS-pentacene/APC. This was due to the differences in the degree of phase separation of each polymer and the TIPS-pentacene. This indicated that the segregation strength between the PS and PαMS was weaker than for APC. This showed that APC is a good polymeric binder candidate for the inkjet printing of TIPS-pentacene as compared to PS and PαMS. The optimized inkjet printing process for TIPS-pentacene/APC OTFTs was used to fabricate OTFTs on a flexible PET substrate. The carrier mobility was 0.27 cm^2^ V^−1^ s^−1^, which was considered to be sufficient for the operation of the electronic display.

A low-voltage OTFT on a plastic substrate was fabricated by Lai et al. [80]. Again, TIPS-pentacene was used as the active channel, which was inkjet-printed using a 16 nozzle cartridge with a volume of 10 pL in a single drop. The solution was prepared at a concentration of 1.5 wt.% using anhydrous anisole as the solvent. Anisole was chosen because of its high boiling point of 153.8 °C, which avoided evaporation before the printing process was completed. The printed organic semiconductor was dried in ambient air and completely dried after 1 min. The fabricated device was able to operate at a low voltage of less than 5 V, with a carrier mobility of 0.22 cm^2^ V^−1^ s^−1^. This study showed that the possibility of using multiple-nozzle inkjet printing on a plastic substrate to fabricate OTFTs and to obtain good carrier mobility. The performance levels of other inkjet-printed OFETs are listed in Table 1 [81].

It can be recognized that the inkjet printing deposition method is a modernized method that provides uniform thin films and potentially well-oriented crystal growth. This is due to the efficient use of the materials and almost complete lack of defects such as pinholes that can be formed on the thin films, as previously reported. Previous studies have revolved more around the concentrations of materials used for the deposition process and the inkjet printer configurations. The inkjet process is costly in terms of its machinery; however, it does allow high-quality thin-film deposition at a faster rate and allows mass production. It is very suitable for use in industrial production sites. There is no material waste when it comes to this deposition method, as it uses the materials efficiently (additive versus subtractive fabrication). There is no limitation on the types of materials used, as one is also able to use polymer blends with this printing method. Flexible substrates have also been studied with this deposition method, indicating its suitability for roll-to-roll fabrication, which could be considerably cheaper.

An important thing to mention is that the rest of the techniques discussed in this manuscript are all blanket deposition approaches, whereas the inkjet method is a “pattern” deposition approach, meaning that the deposition pattern can be produced in specific area with a specific shape, without using a mask or photoresist as the template. This offers a great advantage compared to other deposition methods and potentially reduces the fabrication steps and costs.

Other novel deposition methods are also used, such as flow coating [153], the pen writer deposition method [154], the use of magnetic nanoparticles [155], the spray deposition method [89], the matrix-assisted pulsed laser evaporation (MAPLE) deposition method [156], the roll-to-roll deposition method [156], the gas blow coating deposition method [156], and the blade coating deposition method [156]. These techniques are not covered in this manuscript and do not widely involve pentacene or its derivatives as yet.

## 7. Conclusions

As with many other organic semiconductors, the pentacene has several disadvantages, such as its sensitivity to oxygen, humidity, and light, which could cause defects in the deposited thin-film layers, and hence in the resulting devices. Over the years, researchers have conducted many studies to overcome these issues. One of the outcomes of those studies has been the synthetization of pentacene derivatives, which are separated into two types: pentacene precursors and pentacene substitutes. The precursors allow the use of pentacene in a soluble process and still obtain the pristine pentacene as the desired layer via a heating process. This is achieved by mixing pure pentacene with a chemical additive that turns it into a solution. Once this solution is deposited onto a substrate and has undergone the post-annealing process, the additive will be removed, which leaves behind the original pentacene. However, this technique is not widely used due to the poor characteristics of the pentacene that remains. Therefore, pentacene substitutes are used, which can improve the characteristics of the thin film. This involves a permanent change to the pentacene molecule to improve its viability. This can be seen in its stability during exposure to oxygen, humidity, and light. It has been proven to not be easily damaged by ambient environments, as compared to pristine pentacene. The most studied pentacene substitute is TIPS-pentacene, which has been discussed throughout this manuscript. The importance of the thin-film layer being uniform is well-know and it must have well-oriented crystal growth. The thickness of the thin-film layer also plays an important role in ensuring good flow of the charge carrier. The use of polymer blending also enhances the crystalline growth of this material, which contributes to its performance merits.

This review has compared the various commonly used deposition methods that are associated with pentacene and its derivatives, including the classic thermal vacuum evaporation method, which as the initial method used for thin-film deposition. This approach uses pure pentacene in solid form to produce a uniform thin film, which results in good device performance. However, the disadvantage of this deposition method is the cost of the equipment and materials, as the specified amount of material can only be used in a single deposition process as compared to the soluble-process deposition method, where the same amount can be used for multiple depositions. This limits its production rate and output, as it is performed in a small vacuum chamber. For these reasons, when aiming to achieve low-cost fabrication at mass production rates, soluble process deposition methods are used. The commonly used soluble process deposition method in lab environment is the spin coating process. This is favorable as the cost of the equipment is not as expensive as the equipment used for thermal vacuum evaporation. It also does not require a large volume of source material to be used in its deposition process, which minimizes material waste. Hence, it is suitable for multiple device fabrication on a single substrate by using a small amount of source material. It forms a uniform thin-film layer, which can be enhanced with the use of surface treatments. The crystalline growth of the material in the thin film is also excellent, although the crystal orientation is highly influenced by the centrifugal force during the spinning process. This method has several disadvantages, as the outcome of the deposited layer is uncertain and is vulnerable to chemical reactions with the ambient environment. This issue is overcome by utilizing a glove box in an inert environment. The drop casting method is usually used for deposition on small substrates. It is preferable for the drafting of newly proposed devices and is suitable for flexible and conformable substrates. It has also been shown to produce good crystalline growth due to the small concentrated area involved. Nevertheless, the uniformity of the thin film itself is not guaranteed, as it is influenced by the gravitational force and the surface tension of the solution itself. This is a low-cost deposition method, as it uses only a small volume of material and is not normally used for mass production, which makes it a preferable method when it comes to new device structures or proposals. The dip coating method produces very uniform thin-film layers with good crystalline growth. This method is suitable for deposition on flexible and irregularly shaped substrates. The only downside of this deposition method is that it is not suitable for mass production, as it requires a large reservoir. This is costly and results in a large amount of material waste. Lastly, the inkjet printing method does not focus on the thin-film layer; instead, it forms a uniform and crystalline thin-film layer. This eliminates material waste as it uses the material in an efficient manner. It is suitable to use this method when a change of the device structure is the main variable in the study. Although the equipment is costly, it is well refined for the soluble process method and can also be used for mass production with high production speeds. The inkjet printing technology is mature, and thanks to this it can be integrated with organic electronics. Every deposition method has its specified purposes, and they can be used according to their suitability for the desired device. All of these deposition methods are still being used in ongoing research studies to find ways to improve and enhance their usage.

On a final note, pentacene is not a new material and has been widely studied over the last few decades. Due to its excellent optical and electrical properties, it could have a place in the rising market of wearable and flexible electronics. The synthesis of pentacene derivatives with tunable solubility and optoelectronic properties is the focus of ongoing research. The deposition of solution-based materials pentacene has attracted a lot of attention and opened new doors for the low-cost fabrication of thin-film transistors, memory cards, and circuits for flexible and large-area electronics. Digital printing techniques such as inkjet printing have attracted a lot of industry interest due to their advantages, such as their high speed, low costs, and high precision. The synthesis and preparation of suitable inks for different printing process are key to this process. Other attractive directions for pentacene research based on new applications continue to arise. Pentacene-based OTFTs have been widely studied, although there are still many application opportunities to be developed. For example, for biosensors and bioelectronics, pentacene OTFTs could be used for personal healthcare, the detection of different molecules (protein, ammonia, acetone, ethanol, etc.) closely related with different diseases, or real-time monitoring of strain and stress during sports. Combined with other semiconductor materials such as graphene, tungsten diselenide (WSe2), and zinc oxide (ZnO), pentacene shows promise for application in wide-bandwidth photodetectors and transistors, which have great potential in various other applications, ranging across flexible radio frequency identification tags (RFID), intelligent textiles, smart packaging, imaging, and solar cells.

## Figures and Tables

**Table 1:** Electrical performance comparison of OTFTs based on pentacene and its derivatives.

Reference No.	Deposition Method	Material	Carrier Mobilities (cm^2^ V^−1^ s^−1^)	I_ON/OFF_	Threshold Voltage (V)
[52]	Thermal vacuum evaporation	TMS-pentacene, TES-pentacene, TIPS-pentacene, t-butyl-pentacene, hexyl-pentacene	~10^−5^ ~10^−5^ 0.4 10^−4^ No field conductivity	NIL NIL 10^6^ NIL NIL	NIL
[55]	Thermal vacuum evaporation	Pentacene	0.62	10^2^	−8.5
	Spin coating	Pentacene precursor of 13,6-N-Sulfinylacetamidopentacene (SAP), Pentacene precursor of 6,13-Dihydro-6,13-methanopentacene-15-one (DMP)	0.031 0.09	10^3^	−12.5 −5
[24]	Organic molecular beam	Pentacene	0.435	1.83 × 10^6^	4.77
[57]	Thermal vacuum evaporation	Pentacene	2.5	10^7^	−4
[58]	Thermal vacuum evaporation	Pentacene	0.18	10^3^	NIL
[59]	Thermal vacuum evaporation	Pentacene	0.1	3.3 × 10^5^	1.5
[60]	Thermal vacuum evaporation	Pentacene	0.025	NIL	−1
[61]	Spin coating	TIPS-pentacene	0.002	10^2^	3.2
[62]	Spin coating	TIPS-pentacene	0.005	NIL	−1.3
[63]	Spin coating	TIPS-pentacene	0.6	10^6^	NIL
[64]	Spin coating	TIPS-pentacene	1.66	7 × 10^9^	NIL
[65]	Spin coating	TIPS-pentacene	3.40	10^4^	−10
[66]	Spin coating	TIPS-pentacene	0.05	NIL	NIL
[67]	Drop casting	TIPS-pentacene	0.12	10^4^	−0.2
[68]	Drop casting	TIPS-pentacene	0.005	NIL	5
[69]	Drop casting	TIPS-pentacene	0.00016	NIL	−10
[70]	Drop casting	TIPS-pentacene	0.57	NIL	0.27
[71]	Drop casting	TIPS-pentacene	2.22	1.3 × 10^4^	−5.75
[72]	Drop casting	TIPS-pentacene	0.013	NIL	−0.14
[73]	Drop casting	TIPS-pentacene	0.78	10^4^	−0.56
[74]	Drop casting	TIPS-pentacene	0.15	NIL	NIL
[75]	Drop casting	TIPS-pentacene	0.44	10^5^	−0.3
[76]	Dip coating	TIPS-pentacene	3.0	10^9^	10
[77]	Dip coating	TIPS-pentacene	1.2	NIL	NIL
[39]	Dip coating	TIPS-pentacene	0.047	NIL	NIL
[78]	Inkjet printing	TIPS-pentacene	0.53	1.6 × 10^6^	−0.7
[79]	Inkjet printing	TIPS-pentacene	0.23	2.01 x 10^6^	NIL
[80]	Inkjet printing	TIPS-pentacene	0.22	5.7 × 10^4^	−0.4
[81]	Inkjet printing	TIPS-pentacene	0.35	1.2 × 10^7^	NIL

**Table 2:** Performance comparison of pentacene-based optoelectronic devices.

Reference No.	Deposition Method	Device	Material	Current Efficiency (Cd A^−1^)	Power Efficiency (Im W^−1^)	Turn On Voltage (V)	Quantum Yield (%)	Power Consumption Efficiency (%)
[56]	Thermal vacuum evaporation	OLED	Pentacene	6.6	3.4	5.8	NIL	NIL
[82]	Thermal vacuum evaporation	Solar Cells	Pentacene	NIL	NIL	NIL	NIL	0.33
[86]	Thermal vacuum evaporation	Solar Cells	Pentacene	NIL	NIL	NIL	NIL	5.7
[90]	Thermal vacuum evaporation	OLED	Pentacene	NIL	NIL	5.0	32	NIL
[91]	Thermal vacuum evaporation	OLED	Pentacene	3.4	NIL	NIL	NIL	NIL

## Data Availability

Data sharing is not applicable to this article as no new data were created or analyzed in this study.

## References

[1] Klauk H., Halik M., Zschieschang U., Schmid G., Radlik W., Weber W. (2002). High-mobility polymer gate dielectric pentacene thin film transistors. J. Appl. Phys..

[2] Vets N. (2006). Synthesis of Pentacene Derivatives and Their Application in Organic Thin Film Transistors. Ph.D. Thesis.

[3] Lin Y.-Y., Gundlach D.J., Nelson S.F., Jackson T.N. (1997). Stacked pentacene layer organic thin-film transistors with improved characteristics. IEEE Electron Device Lett..

[4] Park S.K., Jackson T.N., Anthony J.E., Mourey D.A. (2007). High mobility solution processed 6, 13-bis (triisopropyl-silylethynyl) pentacene organic thin film transistors. Appl. Phys. Lett..

[5] Kitamura M., Imada T., Arakawa Y. (2003). Organic light-emitting diodes driven by pentacene-based thin-film transistors. Appl. Phys. Lett..

[6] Wolak M.A., Delcamp J., Landis C.A., Lane P.A., Anthony J., Kafafi Z. (2006). High-Performance Organic Light-Emitting Diodes Based on Dioxolane-Substituted Pentacene Derivatives. Adv. Funct. Mater..

[7] Zhuo M., Chen Y., Sun J., Zhang H., Guo D., Zhang H., Li Q., Wang T., Wan Q. (2013). Humidity sensing properties of a single Sb doped SnO_2_ nanowire field effect transistor. Sens. Actuators B Chem..

[8] Noh Y.-Y., Kim D.-Y. (2007). Organic phototransistor based on pentacene as an efficient red light sensor. Solid-State Electron..

[9] Intaniwet A., Keddie J.L., Shkunov M., Sellin P.J. (2011). High charge-carrier mobilities in blends of poly (triarylamine) and TIPS-pentacene leading to better performing X-ray sensors. Org. Electron..

[10] Ji T., Jung S., Varadan V.K. (2007). Field-controllable flexible strain sensors using pentacene semiconductors. IEEE Electron Device Lett..

[11] Kim J.-M., Jha S.K., Chand R., Lee D.-H., Kim Y.-S. (2011). DNA hybridization sensor based on pentacene thin film transistor. Biosens. Bioelectron..

[12] Azeman N.H., Ahmad Khusaini M.A., Daik R., Ismail A.G., Yeop Majlis B., Mat Salleh M.T.H., Tg Abdul Aziz T.H., A Bakar A.A., Md Zain A.R., Teh C.-H. (2021). Synthesis of a novel 1, 4-bis [2-(5-thiophen-2-yl)-1-benzothiophene]-2, 5-dioctyloxybenzene pentamer for creatinine detection. Asian J. Org. Chem..

[13] Nashruddin S.N.A., Abdullah J., Mohammad Haniff M.A.S., Mat Zaid M.H., Choon O.P., Mohd Razip Wee M.F. (2021). Label Free Glucose Electrochemical Biosensor Based on Poly(3,4-ethylenedioxy thiophene):Polystyrene Sulfonate/Titanium Carbide/Graphene Quantum Dots. Biosensors.

[14] Wan Khalid W.E.F., Heng L.Y., Mat Arip M.N. (2018). Surface Modification of Cellulose Nanomaterial for Urea Biosensor Application. Sains Malays..

[15] Boukhili W., Mahdouani M., Erouel M., Puigdollers J., Bourguiga R. (2015). Reversibility of humidity effects in pentacene based organic thin-film transistor: Experimental data and electrical modeling. Synth. Met..

[16] Tang Q., Zhang D., Wang S., Ke N., Xu J., Yu J.C., Miao Q. (2009). A meaningful analogue of pentacene: Charge transport, polymorphs, and electronic structures of dihydrodiazapentacene. Chem. Mater..

[17] Bhatia R., Wadhawa D., Gurtu G., Gaur J., Gupta D. (2019). Methodologies for the synthesis of pentacene and its derivatives. J. Saudi Chem. Soc..

[18] Kim H.G., Choi H.H., Song E., Cho K., Choi E.-J. (2015). Synthesis, stability and electrical properties of new soluble pentacenes with unsaturated side groups. RSC Adv..

[19] Benor A., Hoppe A., Wagner V., Knipp D. (2007). Electrical stability of pentacene thin film transistors. Org. Electron..

[20] Herwig P.T., Müllen K. (1999). A soluble pentacene precursor: Synthesis, solid-state conversion into pentacene and application in a field-effect transistor. Adv. Mater..

[21] Weidkamp K.P., Afzali A., Tromp R.M., Hamers R.J. (2004). A photopatternable pentacene precursor for use in organic thin-film transistors. J. Am. Chem. Soc..

[22] Takahashi T., Kitamura M., Shen B., Nakajima K. (2000). Straightforward method for synthesis of highly alkyl-substituted naphthacene and pentacene derivatives by homologation. J. Am. Chem. Soc..

[23] Anthony J.E., Brooks J.S., Eaton D.L., Parkin S.R. (2001). Functionalized pentacene: Improved electronic properties from control of solid-state order. J. Am. Chem. Soc..

[24] Lee T., Lim B., Yong K., Kwon W., Park M. (2017). Effects of oxygen plasma generated in magnetron sputtering of ruthenium oxide on pentacene thin film transistors. Korean J. Chem. Eng..

[25] Qu H., Shen H., Li J., Men Y., Chen X., Chong Z. (2018). Synthesis and characterization of three pentacene derivatives. Trans. Tianjin Univ..

[26] Zhao Y., Yan L., Murtaza I., Liang X., Meng H., Huang W. (2017). A thermally stable anthracene derivative for application in organic thin film transistors. Org. Electron..

[27] Brega V., Yan Y., Thomas S.W. (2020). Acenes beyond organic electronics: Sensing of singlet oxygen and stimuli-responsive materials. Org. Biomol. Chem..

[28] Knipp D., Benor A., Wagner V., Muck T. (2007). Influence of impurities and structural properties on the device stability of pentacene thin film transistors. J. Appl. Phys..

[29] Rincon-Llorente G. (2010). Synthesis and Characterisation of Solution Processable Acenes.

[30] Nawrocki R.A. (2019). Super-and Ultrathin Organic Field-Effect Transistors: From Flexibility to Super-and Ultraflexibility. Adv. Funct. Mater..

[31] Glynn C., O’Dwyer C. (2017). Solution processable metal oxide thin film deposition and material growth for electronic and photonic devices. Adv. Mater. Interfaces.

[32] Deng X., Xiong F., Li X., Xiang B., Li Z., Wu X., Guo C., Li X., Li Y., Li G. (2018). Application of atomic force microscopy in cancer research. J. Nanobiotechnol..

[33] Assadpour E., Rostamabadi H., Jafari S.M. (2020). Introduction to characterization of nanoencapsulated food ingredients. Characterization of Nanoencapsulated Food Ingredients.

[34] Pérez-Gutiérrez E., Cerón M., Santos P., Ceballos P., Venkatesan P., Thamotharan S., Bernal-Pinilla W., Barbosa-García O., Percino M.J. (2019). Film morphology of acrylonitrile materials deposited by a solution process and vacuum evaporation. Supramolecular interactions, optoelectronic properties and an approximation by computational calculations. New J. Chem..

[35] Widjonarko N.E. (2016). Introduction to advanced X-ray diffraction techniques for polymeric thin films. Coatings.

[36] Rider A.N., Arnott D.R. (2000). Boiling water and silane pre-treatment of aluminum alloys for durable adhesive bonding. Int. J. Adhes. Adhes..

[37] Alias A.N., Zabidi Z.M., Ali A.M.M., Harun M.K., Yahya M.Z.A. (2013). Optical characterization and properties of polymeric materials for optoelectronic and photonic applications. Int. J. Appl. Sci. Technol..

[38] Aziz S.B., Abdullah O.G., Hussein A.M., Abdulwahid R.T., Rasheed M.A., Ahmed H.M., Abdalqadir S.W., Mohammed A.R. (2017). Optical properties of pure and doped PVA: PEO based solid polymer blend electrolytes: Two methods for band gap study. J. Mater. Sci. Mater. Electron..

[39] Yoshimoto S., Takahashi K., Suzuki M., Yamada H., Miyahara R., Mukai K., Yoshinobu J. (2017). Highly anisotropic mobility in solution processed TIPS-pentacene film studied by independently driven four GaIn probes. Appl. Phys. Lett..

[40] Newman C.R., Chesterfield R.J., Panzer M.J., Frisbie C.D. (2005). High mobility top-gated pentacene thin-film transistors. J. Appl. Phys..

[41] Katharria Y.S., Kumar S., Prakash R., Choudhary R.J., Singh F., Phase D.M., Kanjilal D. (2007). Characterizations of pulsed laser deposited SiC thin films. J. Non. Cryst. Solids.

[42] Sneh O., Clark-Phelps R.B., Londergan A.R., Winkler J., Seidel T.E. (2002). Thin film atomic layer deposition equipment for semiconductor processing. Thin Solid Films.

[43] Tétard F., Djemia P., Besland M.P., Tessier P.Y., Angleraud B. (2005). Characterizations of CNx thin films made by ionized physical vapor deposition. Thin Solid Films.

[44] Sun Y., Liu Y., Zhu D. (2005). Advances in organic field-effect transistors. J. Mater. Chem..

[45] Dey A., Singh A., Das D., Iyer P.K. (2015). Organic Semiconductors: A new future of nanodevices and applications. Thin Film Structures in Energy Applications.

[46] Kowarik S., Gerlach A., Schreiber F. (2008). Organic molecular beam deposition: Fundamentals, growth dynamics, and in situ studies. J. Phys. Condens. Matter.

[47] Wit B. Defects in Pentacene Thin Films Grown by Supersonic Molecular Beam Deposition.

[48] Gong X., Xin M., Li M., Yuan H., Xie J. (2017). Compatibilizer improving properties of tea dust/polylactic acid biomass composites. Nongye Gongcheng Xuebao Trans. Chin. Soc. Agric. Eng..

[49] Xu J., Zhu X., Tan S., Zhang Y., Li B., Tian Y., Shan H., Cui X., Zhao A., Dong Z. (2021). Determining structural and chemical heterogeneities of surface species at the single-bond limit. Science.

[50] Navarro F.F., Djurovich P.I., Thompson M.E. (2014). Metal deposition for optoelectronic devices using a low vacuum vapor phase deposition (VPD) system. Org. Electron..

[51] Burrows P.E., Forrest S.R., Sapochak L.S., Schwartz J., Fenter P., Buma T., Ban V.S., Forrest J.L. (1995). Organic vapor phase deposition: A new method for the growth of organic thin films with large optical non-linearities. J. Cryst. Growth.

[52] Sheraw C.D., Jackson T.N., Eaton D.L., Anthony J.E. (2003). Functionalized pentacene active layer organic thin-film transistors. Adv. Mater..

[53] Ismail A.G., Hill I.G. (2011). Stability of n-channel organic thin-film transistors using oxide, SAM-modified oxide and polymeric gate dielectrics. Org. Electron. Phys. Mater. Appl..

[54] Ismail A.G. (2018). The Impact of Alkyl Phosphonic Acid Monolayer Modified Dielectrics on the Performance of n-Channel Organic Thin-Film Transistors. Mater. Focus.

[55] Ochiai S., Palanisamy K., Kannappan S., Shin P.-K. (2012). Pentacene active channel layers prepared by spin-coating and vacuum evaporation using soluble precursors for OFET applications. Int. Sch. Res. Netw. Condens. Matter Phys..

[56] Saikia D., Sarma R. (2016). Fabrication and Characterization of Organic Light Emitting Diode Using FTO/Pentacene as Bilayer Anode. Mater. Sci. Ind. J..

[57] Liao K., Ismail A.G., Kreplak L., Schwartz J., Hill I.G. (2010). Designed Organophosphonate Self-Assembled Monolayers Enhance Device Performance of Pentacene-Based Organic Thin-Film Transistors. Adv. Mater..

[58] Yang C.-Y., Cheng S.-S., Ou T.-M., Wu M.-C., Wu C.-H., Chao C.-H., Lin S.-Y., Chan Y.-J. (2007). Pentacene-based planar-and vertical-type organic thin-film transistor. IEEE Trans. Electron Devices.

[59] Guo E., Xing S., Dollinger F., Wu Z., Tahn A., Löffler M., Leo K., Kleemann H. (2020). High-Performance Static Induction Transistors Based on Small-Molecule Organic Semiconductors. Adv. Mater. Technol..

[60] Hiroki M., Maeda Y., Ohmi S. (2018). Top-gate pentacene-based organic field-effect transistor with amorphous rubrene gate insulator. Jpn. J. Appl. Phys..

[61] Da Silva Ozório M., Nogueira G.L., Morais R.M., da Silva Martin C., Constantino C.J.L., Alves N. (2016). Poly (3-hexylthiophene): TIPS-pentacene blends aiming transistor applications. Thin Solid Films.

[62] Ozório M.S., Camacho S.A., Cordeiro N.J.A., Duarte J.L., Alves N. (2018). Solvent Effect on Morphology and Optical Properties of Poly (3-hexylthiophene): TIPS-Pentacene Blends. J. Electron. Mater..

[63] Birnie D.P. (2013). A model for drying control cosolvent selection for spin-coating uniformity: The thin film limit. Langmuir.

[64] Wang S., Zhao X., Tong Y., Tang Q., Liu Y. (2020). Directly Spin Coating a Low-Viscosity Organic Semiconductor Solution onto Hydrophobic Surfaces: Toward High-Performance Solution-Processable Organic Transistors. Adv. Mater. Interfaces.

[65] Yoo H., Choi H.H., Shin T.J., Rim T., Cho K., Jung S., Kim J. (2015). Self-Assembled, Millimeter-Sized TIPS-Pentacene Spherulites Grown on Partially Crosslinked Polymer Gate Dielectric. Adv. Funct. Mater..

[66] Kim H.S., Park J.H., Lee W.H., Kim H.H., Park Y.D. (2019). Tailoring the crystallinity of solution-processed 6, 13-bis (triisopropylsilylethynyl) pentacene via controlled solidification. Soft Matter.

[67] Raghuwanshi V., Bharti D., Tiwari S.P. (2016). Flexible organic field-effect transistors with TIPS-Pentacene crystals exhibiting high electrical stability upon bending. Org. Electron..

[68] Shih A., Akinwande A.I. (2017). Solution-processed high-voltage organic thin film transistor. MRS Adv..

[69] Shih A., Akinwande A.I. (2018). Solution-Processed and Self-Assembled Monolayer-Treated High-Voltage Organic Thin Film Transistors for Flexible MEMS Integration. MRS Adv..

[70] Raghuwanshi V., Bharti D., Mahato A.K., Varun I., Tiwari S.P. (2018). Semiconductor: Polymer blend ratio dependent performance and stability in low voltage flexible organic field-effect transistors. Synth. Met..

[71] Zhou Y., Wang H., Tang Q., Tong Y., Zhao X., Liu Y. (2018). Solution-Processed Single-Crystal Array for High-Performance Conformable Transistors. IEEE Electron Device Lett..

[72] Lago N., Buonomo M., Imran S., Bertani R., Wrachien N., Bortolozzi M., Pedersen M.G., Cester A. (2018). TIPS-pentacene as biocompatible material for solution processed high-performance electronics operating in water. IEEE Electron Device Lett..

[73] Raghuwanshi V., Bharti D., Mahato A.K., Varun I., Tiwari S.P. (2019). TIPS-Pentacene: PS Blend Organic Field-Effect Transistors with Hybrid Gate Dielectric on Paper Substrate. Proceedings of the 2019 International Symposium on VLSI Technology, Systems and Application (VLSI-TSA).

[74] He Z., Zhang Z., Bi S., Asare-Yeboah K., Chen J., Li D. (2020). A facile and novel route to improve TIPS pentacene based organic thin film transistor performance with elastomer. Synth. Met..

[75] Raghuwanshi V., Bharti D., Varun I., Mahato A.K., Tiwari S.P. (2016). Performance enhancement in mechanically stable flexible organic-field effect transistors with TIPS-pentacene: Polymer blend. Org. Electron..

[76] Wang W., Wang L., Dai G., Deng W., Zhang X., Jie J., Zhang X. (2017). Controlled growth of large-area aligned single-crystalline organic nanoribbon arrays for transistors and light-emitting diodes driving. Nano-Micro Lett..

[77] Manaka T., Iwamoto M. (2018). Spectroscopic Imaging for Studying Carrier Behaviour in Organic Semiconductor Thin Films. Proceedings of the 2018 12th International Conference on Advanced Semiconductor Devices and Microsystems (ASDAM).

[78] Cho S.Y., Ko J.M., Lim J., Lee J.Y., Lee C. (2013). Inkjet-printed organic thin film transistors based on TIPS pentacene with insulating polymers. J. Mater. Chem. C.

[79] Kim J.S., Song C.K. (2015). Patterning process of ink-jet printed 6, 13-bis (triisopropylsilylethynyl) pentacene layer using bank structures for organic thin film transistors. Thin Solid Films.

[80] Lai S., Cosseddu P., Zucca A., Loi A., Bonfiglio A. (2017). Combining inkjet printing and chemical vapor deposition for fabricating low voltage, organic field-effect transistors on flexible substrates. Thin Solid Films.

[81] Ge F., Wang X., Zhang Y., Song E., Zhang G., Lu H., Cho K., Qiu L. (2017). Modulating the Surface via Polymer Brush for High-Performance Inkjet-Printed Organic Thin-Film Transistors. Adv. Electron. Mater..

[82] Biber M., Aydoğan Ş., Çaldıran Z., Çakmak B., Karacalı T., Türüt A. (2017). The influence of annealing temperature and time on the efficiency of pentacene: PTCDI organic solar cells. Results Phys..

[83] Zomerman D., Kong J., McAfee S.M., Welch G.C., Kelly T.L. (2018). Control and Characterization of Organic Solar Cell Morphology Through Variable-Pressure Solvent Vapor Annealing. Am. Chem. Soc. Appl. Energy Mater..

[84] Chen W., Nikiforov M.P., Darling S.B. (2012). Morphology characterization in organic and hybrid solar cells. Energy Environ. Sci..

[85] Pan J., Zhang X., Zheng Y., Xiang W. (2021). Morphology control of perovskite film for efficient CsPbIBr2 based inorganic perovskite solar cells. Sol. Energy Mater. Sol. Cells.

[86] Zhang X., Li M., Dall’Agnese C., Chen G., Wang X.-F., Miyasaka T. (2019). Thermo-evaporated pentacene and perylene as hole transport materials for perovskite solar cells. Dyes Pigments.

[87] Lubert-Perquel D., Kim D.K., Robaschik P., Kay C.W.M., Heutz S. (2019). Growth, morphology and structure of mixed pentacene films. J. Mater. Chem. C.

[88] Zhang K., Marszalek T., Wucher P., Wang Z., Veith L., Lu H., Räder H., Beaujuge P.M., Blom P.W.M., Pisula W. (2018). Crystallization Control of Organic Semiconductors during Meniscus-Guided Coating by Blending with Polymer Binder. Adv. Funct. Mater..

[89] Onojima N., Obata S., Nakamura A., Hara K. (2017). Influence of phase-separated morphology on small molecule/polymer blend organic field-effect transistors fabricated using electrostatic spray deposition. Thin Solid Films.

[90] Wolak M.A., Jang B.-B., Palilis L.C., Kafafi Z.H. (2004). Functionalized pentacene derivatives for use as red emitters in organic light-emitting diodes. J. Phys. Chem. B.

[91] Rao M.V.M., Huang T.-S., Su Y.-K., Huang Y.-T. (2009). Fullerene and pentacene as a pure organic connecting layer in tandem organic light emitting devices. J. Electrochem. Soc..

[92] Lassnig R., Hollerer M., Striedinger B., Fian A., Stadlober B., Winkler A. (2015). Optimizing pentacene thin-film transistor performance: Temperature and surface condition induced layer growth modification. Org. Electron..

[93] Ahn T., Choi Y., Yi M.H. (2008). A new approach to the surface modification of polymeric gate insulators for organic thin-film transistor applications. Appl. Surf. Sci..

[94] Kleemann H., Schuenemann C., Zakhidov A.A., Riede M., Lüssem B., Leo K. (2012). Structural phase transition in pentacene caused by molecular doping and its effect on charge carrier mobility. Org. Electron..

[95] Ismail A.G. (2018). Photolithographically patterned N-channel organic thin film transistors using sensitized polyvinyl alcohol. Org. Electron..

[96] Van Franeker J.J., Hermida-Merino D., Gommes C., Arapov K., Michels J.J., Janssen R.A.J., Portale G. (2017). Sub-Micrometer Structure Formation during Spin Coating Revealed by Time-Resolved In Situ Laser and X-Ray Scattering. Adv. Funct. Mater..

[97] Han D.H., Kim D., Yun H.W., Lee J., Lee U.G., Chung H.S., Kim W.-B. (2021). Effects of aging on the thickness of a homogeneous film fabricated using a spin coating process. J. Coat. Technol. Res..

[98] Lee U.G., Kim W.-B., Han D.H., Chung H.S. (2019). A Modified Equation for Thickness of the Film Fabricated by Spin Coating. Symmetry.

[99] Rueda-Delgado D., Hossain I.M., Jakoby M., Schwenzer J.A., Abzieher T., Howard I.A., Richards B.S., Lemmer U., Paetzold U.W. (2020). Solution-processed and evaporated C60 interlayers for improved charge transport in perovskite photovoltaics. Org. Electron..

[100] Dissanayake N., Abeysundara S., Wanasekara N.D. (2020). Investigating the Feasibility of Applying Spin Coating Method for Textiles. Proceedings of the 2020 Moratuwa Engineering Research Conference (MERCon).

[101] Bharti D., Tiwari S.P. (2015). Improved alignment and crystallinity of TIPS-Pentacene thin films by off-center spin coating. Proceedings of the 2015 IEEE 15th International Conference on Nanotechnology (IEEE-NANO).

[102] Akkerman H.B., Li H., Bao Z. (2012). TIPS-pentacene crystalline thin film growth. Org. Electron..

[103] He Z. (2014). Tips Pentacene Crystal Alignment for Improving Performance of Solution Processed Organic Thin Film Transistors. Ph.D. Thesis.

[104] Xiao C., Kan X., Liu C., Jiang W., Zhao G., Zhao Q., Zhang L., Hu W., Wang Z., Jiang L. (2017). Controlled formation of large-area single-crystalline TIPS-pentacene arrays through superhydrophobic micropillar flow-coating. J. Mater. Chem. C.

[105] Madec M.-B., Smith P.J., Malandraki A., Wang N., Korvink J.G., Yeates S.G. (2010). Enhanced reproducibility of inkjet printed organic thin film transistors based on solution processable polymer-small molecule blends. J. Mater. Chem..

[106] Lee M.W., Ryu G.S., Lee Y.U., Pearson C., Petty M.C., Song C.K. (2012). Control of droplet morphology for inkjet-printed TIPS-pentacene transistors. Microelectron. Eng..

[107] Nagarjuna G., Baghgar M., Labastide J.A., Algaier D.D., Barnes M.D., Venkataraman D. (2012). Tuning aggregation of poly (3-hexylthiophene) within nanoparticles. Am. Chem. Soc. Nano.

[108] Jung S.-B., Ha T.-J., Park H.-H. (2007). Roughness and pore structure control of ordered mesoporous silica films for the enhancement of electrical properties. J. Appl. Phys..

[109] Qian J., Jiang S., Li S., Wang X., Shi Y., Li Y. (2019). Solution-Processed 2D Molecular Crystals: Fabrication Techniques, Transistor Applications, and Physics. Adv. Mater. Technol..

[110] Liu D., Xu X., Su Y., He Z., Xu J., Miao Q. (2013). Self-Assembled Monolayers of Phosphonic Acids with Enhanced Surface Energy for High-Performance Solution-Processed N-Channel Organic Thin-Film Transistors. Angew. Chem..

[111] Zhang Z., Peng B., Ji X., Pei K., Chan P.K.L. (2017). Marangoni-effect-assisted bar-coating method for high-quality organic crystals with compressive and tensile strains. Adv. Funct. Mater..

[112] Zhang F., Di C., Berdunov N., Hu Y., Hu Y., Gao X., Meng Q., Sirringhaus H., Zhu D. (2013). Ultrathin film organic transistors: Precise control of semiconductor thickness via spin-coating. Adv. Mater..

[113] Asiri A.M., Chani M.T.S., Khan S.B. (2018). Method of Making Thin Film Humidity Sensors. U.S. Patent.

[114] Eslamian M. (2017). Inorganic and organic solution-processed thin film devices. Nano-Micro Lett..

[115] Lee T.D., Ebong A.U. (2017). A review of thin film solar cell technologies and challenges. Renew. Sustain. Energy Rev..

[116] Park J.-M., Sohn I.-B., Kang C., Kee C.-S., Hwang I.-W., Yoo H.K., Lee J.W. (2016). Terahertz modulation using TIPS-pentacene thin films deposited on patterned silicon substrates. Opt. Commun..

[117] Patrick D.L., Schaaf C., Morehouse R., Johnson B.L. (2019). Multi-scale modeling of early-stage morphology in solution-processed polycrystalline thin films. Phys. Chem. Chem. Phys..

[118] He Z., Zhang Z., Bi S. (2019). Small-molecule additives for organic thin film transistors. J. Mater. Sci. Mater. Electron..

[119] Hakeem A., Murtaza G. (2017). Structural, optical, electrochemical, thermal and electrical properties of 6, 13-bis (tri-isopropylsilylethynyl) tips-pentacene. Dig. J. Nanomater. Biostructures.

[120] Pipan G., Bogar M., Ciavatti A., Basiricò L., Cramer T., Fraboni B., Fraleoni-Morgera A. (2018). Direct Inkjet Printing of TIPS-Pentacene Single Crystals onto Interdigitated Electrodes by Chemical Confinement. Adv. Mater. Interfaces.

[121] Asare–Yeboah K., Bi S., He Z., Li D. (2016). Temperature gradient controlled crystal growth from TIPS pentacene-poly (α-methyl styrene) blends for improving performance of organic thin film transistors. Org. Electron..

[122] Zissis G., Bertoldi P., Serrenho T. (2021). Update on the Status of LED-Lighting World Market Since 2018.

[123] Olcer Y.A., Tascon M., Eroglu A.E., Boyacı E. (2019). Thin film microextraction: Towards faster and more sensitive microextraction. TrAC Trends Anal. Chem..

[124] Hilliard S., Baldinozzi G., Friedrich D., Kressman S., Strub H., Artero V., Laberty-Robert C. (2017). Mesoporous thin film WO3 photoanode for photoelectrochemical water splitting: A sol–gel dip coating approach. Sustain. Energy Fuels.

[125] Miao S.S., Wu M.S., Ma L.Y., He X.J., Yang H. (2016). Electrochemiluminescence biosensor for determination of organophosphorous pesticides based on bimetallic Pt-Au/multi-walled carbon nanotubes modified electrode. Talanta.

[126] Wu K., Li H., Li L., Zhang S., Chen X., Xu Z., Zhang X., Hu W., Chi L., Gao X. (2016). Controlled growth of ultrathin film of organic semiconductors by balancing the competitive processes in dip-coating for organic transistors. Langmuir.

[127] Liu X., Zhang Y., Zhang X., Li R., Hu W. (2020). Continuous and highly ordered organic semiconductor thin films via dip-coating: The critical role of meniscus angle. Sci. China Mater..

[128] Ravariu C., Mihaiescu D., Morosan A., Vasile B.S., Purcareanu B. (2020). Sulpho-Salicylic Acid Grafted to Ferrite Nanoparticles for n-Type Organic Semiconductors. Nanomaterials.

[129] Morosan A., Mihaiescu D.E., Istrati D., Voicu G., Fudulu A., Stan R. (2018). Polar shell magnetic nanostructured systems for heterogeneous nanophase reactions. UPB Sci. Bull. Ser. B.

[130] Chen F.-C., Kung L.-J., Chen T.-H., Lin Y.-S. (2007). Copper phthalocyanine buffer layer to enhance the charge injection in organic thin-film transistors. Appl. Phys. Lett..

[131] Di C., Yu G., Liu Y., Guo Y., Wang Y., Wu W., Zhu D. (2008). High-performance organic field-effect transistors with low-cost copper electrodes. Adv. Mater..

[132] Zhang D.K., Liu Y.C., Liu Y.L., Yang H. (2004). The electrical properties and the interfaces of Cu_2_O/ZnO/ITO p–i–n heterojunction. Phys. B Condens. Matter.

[133] Yang T., Mehta J.S., Haruk A.M., Mativetsky J.M. (2018). Targeted deposition of organic semiconductor stripes onto rigid, flexible, and three-dimensional substrates. J. Mater. Chem. C.

[134] Jang J., Nam S., Im K., Hur J., Cha S.N., Kim J., Son H.B., Suh H., Loth M.A., Anthony J.E. (2012). Highly crystalline soluble acene crystal arrays for organic transistors: Mechanism of crystal growth during dip-coating. Adv. Funct. Mater..

[135] Bittle E.G., Basham J.I., Jackson T.N., Jurchescu O.D., Gundlach D.J. (2016). Mobility overestimation due to gated contacts in organic field-effect transistors. Nat. Commun..

[136] Kanagawa T., Hobara R., Matsuda I., Tanikawa T., Natori A., Hasegawa S. (2003). Anisotropy in conductance of a quasi-one-dimensional metallic surface state measured by a square micro-four-point probe method. Phys. Rev. Lett..

[137] Zhou Z., Cantu L.R., Chen X., Alexander M.R., Roberts C.J., Hague R., Tuck C., Irvine D., Wildman R. (2019). High-throughput characterization of fluid properties to predict droplet ejection for three-dimensional inkjet printing formulations. Addit. Manuf..

[138] Winoto A. (2014). Methods and Compositions for Ink Jet Deposition of Conductive Features. U.S. Patent.

[139] Mościcki A., Fałat T., Smolarek A., Kinart A., Felba J., Borecki J. (2012). Interconnection process by ink jet printing method. Proceedings of the 2012 12th IEEE International Conference on Nanotechnology (IEEE-NANO).

[140] Mikami Y., Yoshioka H., Oki Y. (2021). Fully room temperature and label free biosensing based on an ink-jet printed polymer microdisk laser. Opt. Mater. Express.

[141] Muniz N.O., Vechietti F.A., dos Santos L.A.L. (2019). Influence of several binders on the mechanical properties of alumina parts manufactured by 3D inkjet printing. Mater. Res. Express.

[142] Jeong S., Kim D., Moon J. (2008). Ink-Jet-printed organic—Inorganic hybrid dielectrics for organic thin-film transistors. J. Phys. Chem. C.

[143] Wen Y.X., Liu S.G., Tao B.X., Luo H.Q., Li N.B. (2020). A signal-off photocathode biosensor based on a novel metal-organic polymer for the detection of glucose. Sens. Actuators B Chem..

[144] Xiang C., Wu L., Lu Z., Li M., Wen Y., Yang Y., Liu W., Zhang T., Cao W., Tsang S.-W. (2020). High efficiency and stability of ink-jet printed quantum dot light emitting diodes. Nat. Commun..

[145] Zhang G., Zhang P., Chen H., Guo T. (2018). Modification of polymer gate dielectrics for organic thin-film transistor from inkjet printing. Appl. Phys. A.

[146] Kwon Y.-J., Park Y.D., Lee W.H. (2016). Inkjet-printed organic transistors based on organic semiconductor/insulating polymer blends. Materials.

[147] Li X., Smaal W.T.T., Kjellander C., van der Putten B., Gualandris K., Smits E.C.P., Anthony J., Broer D.J., Blom P.W.M., Genoe J. (2011). Charge transport in high-performance ink-jet printed single-droplet organic transistors based on a silylethynyl substituted pentacene/insulating polymer blend. Org. Electron..

[148] James D.T., Kjellander B.K.C., Smaal W.T.T., Gelinck G.H., Combe C., McCulloch I., Wilson R., Burroughes J.H., Bradley D.D.C., Kim J.-S. (2011). Thin-film morphology of inkjet-printed single-droplet organic transistors using polarized Raman spectroscopy: Effect of blending TIPS-pentacene with insulating polymer. Am. Chem. Soc. Nano.

[149] Flory P.J. (1942). Thermodynamics of high polymer solutions. J. Chem. Phys..

[150] Huggins M.L. (1942). The viscosity of dilute solutions of long-chain molecules. IV. Dependence on concentration. J. Am. Chem. Soc..

[151] Michels J.J. (2011). Surface-Directed Spinodal Decomposition of Solvent-Quenched Organic Transistor Blends. ChemPhysChem.

[152] Lim J.A., Lee H.S., Lee W.H., Cho K. (2009). Control of the morphology and structural development of solution-processed functionalized acenes for high-performance organic transistors. Adv. Funct. Mater..

[153] Matsuda Y., Nakahara Y., Michiura D., Uno K., Tanaka I. (2016). High-mobility 6, 13-bis (triisopropylsilylethynyl) pentacene transistors using solution-processed polysilsesquioxane gate dielectric layers. J. Nanosci. Nanotechnol..

[154] Li Y., Wan J., Smilgies D.-M., Bouffard N., Sun R., Headrick R.L. (2016). Nucleation and strain-stabilization during organic semiconductor thin film deposition. Sci. Rep..

[155] Kwon J.-H., Kim D.-K., Jang J., Park J., Kang S.-W., Bae J.-H. (2017). Self-alignment of 6, 13-bis (triisopropylsilylethynyl) pentacene molecules through magnetic flux-affected nanoparticle motion in solution-processed transistors. Org. Electron..

[156] Zhang X., Deng W., Lu B., Fang X., Zhang X., Jie J. (2020). Fast deposition of an ultrathin, highly crystalline organic semiconductor film for high-performance transistors. Nanoscale Horiz..

